# Exploring the Dynamic Relationship between the Gut Microbiome and Body Composition across the Human Lifespan: A Systematic Review

**DOI:** 10.3390/nu16050660

**Published:** 2024-02-26

**Authors:** Ifigeneia Komodromou, Eleni Andreou, Angelos Vlahoyiannis, Maria Christofidou, Kyriacos Felekkis, Myrtani Pieri, Christoforos D. Giannaki

**Affiliations:** 1 Department of Life Sciences, School of Life and Health Sciences, University of Nicosia, 2417 Nicosia, Cyprus; ifigeniakomo@gmail.com (I.K.); andreou.el@unic.ac.cy (E.A.); vlahoyiannis.a@unic.ac.cy (A.V.); christofidou.m@unic.ac.cy (M.C.); felekkis.k@unic.ac.cy (K.F.); pieri.m@unic.ac.cy (M.P.); 2 Research Centre for Exercise and Nutrition (RECEN), 2417 Nicosia, Cyprus

**Keywords:** gut microbiota, obesity, overweight, body fat, fat mass, muscle mass

## Abstract

This systematic review aimed to identify different gut microbiome profiles across the human lifespan and to correlate such profiles with the body composition. PubMed, Scopus, and Cochrane were searched from inception to March 2022. Sixty studies were included in this systematic review. Overall, the gut microbiome composition in overweight participants exhibited decreased α-diversity, decreased levels of the phylum *Bacteroidetes* and its taxa, and increased levels of the phylum *Firmicutes*, its taxa, and the *Firmicutes*/*Bacteroidetes* ratio, in comparison to normal-weight participants. Other body composition parameters showed similar correlations. Fat mass and waist circumference were found to correlate positively with the *Firmicutes* taxa and negatively with the *Bacteroidetes* taxa. In contrast, lean body mass and muscle mass demonstrated a positive correlation with the *Bacteroidetes* taxa. Notably, these correlations were more pronounced in athletes than in obese and normal-weight individuals. The composition of the gut microbiome is evidently different in overweight individuals or athletes of all age groups, with the former tending towards decreased *Bacteroidetes* taxa and increased *Firmicutes* taxa, while a reversed relationship is observed concerning athletes. Further studies are needed to explore the dynamic relationship between energy intake, body composition, and the gut microbiome across the human lifespan.

## 1. Introduction

The gut microbiome is involved in multiple essential functions responsible for the normal functioning of the intestine and the host [1,2], but its composition is unique to each person. In fact, there is less than 10% similarity between any two individuals [3]. Its formation is determined early from birth through adulthood and modified by genetic and environmental factors, such as diet, physical activity, age, gender, sleep, smoking, and antibiotics [1,4].

The brain participates dynamically in energy balance regulation via its ability to communicate with the peripheral organs through various nerve and chemical signals, most of them coming from the gastrointestinal tract, a relationship called the gut–brain axis [5]. The activation of neuropeptide Y/agouti-related peptide (NPY/AGRP) neurons in the hypothalamus of the brain by the hormone ghrelin has an orexigenic effect by stimulating an increase in appetite and a decrease in energy expenditure [6,7]. Ghrelin is negatively correlated with the genera *Bifidobacterium*, *Lactobacillus*, *Blautia coccoides*, and *Eubacterium rectale* and positively correlated with the genera *Bacteroides* and *Prevotella* [8,9]. The activation of pro-opiomelanocortin/cocaine-amphetamine-related transcript (POMC/CART) and the suppression of NPY/AGRP neurons by the hormones insulin, leptin, cholecystokinin (CCK), peptide YY (PYY), glucagon-like-peptide 1 (GLP-1), and oxyntomodulin (OXM) leads to the opposite, anorexigenic effects [6,7].

Moreover, the specific pathways through which the gut microbiome communicates with the brain and interacts with energy expenditure, body weight, and body composition are well known. The first mechanism involves lipopolysaccharides (LPS), found in the cell walls of Gram-negative bacteria on macrophages and adipose tissue. LPS activate a cascade of pro-inflammatory responses that are accountable for a chronic state of underlying inflammation [8,9,10,11,12,13]. The second mechanism involves short-chain fatty acids (SCFAs), which are produced by the fermentation of fiber. In addition to contributing to approximately 10% of energy intake, they participate in other metabolic pathways, such as promoting hepatic lipogenesis and gluconeogenesis, inflammatory reduction, and an increase in GLP-1 and PYY production [8,10,11,12]. The latter mechanism involves bile acids, which are fermented by the colon microbiome to produce secondary bile acids. Secondary bile acids later promote increased energy expenditure and the production of GLP-1 [8].

As a result, the gut microbiome profile appears to be different in overweight and obese compared to normal-weight individuals, demonstrated through various studies [14,15,16]. Dysbiosis, or the imbalance of the gut microbiota, has been associated with inflammatory responses observed in clinical conditions, underlying the microbiota’s influence on gastrointestinal health and disease mechanisms [17]. Recent findings underscore the potential of monitoring the gut microbiome for diagnostic and therapeutic strategies for inflammatory bowel diseases (IBD), irritable bowel syndrome (IBS), and colorectal cancer, highlighting the microbiota’s integral role in gastrointestinal health [18]. Notably, it is suggested that gut microbiome modification could be a potential strategy for the early prevention and treatment of relevant conditions and obesity, through improving dietary habits; taking probiotics, prebiotics, and synbiotics; and fecal microbiota transplantation from healthy individuals [10,19,20].

However, based on the literature review, the formation of the gut microbiome in relationship to the body composition is poorly systematized. Specifically, there are no summarized gut microbiome profiles across the human lifespan according to age groups in healthy individuals. Thus, the current systematic review focused on identifying different gut microbiome profiles in healthy individuals, from children to older adults, and to correlate such profiles with the body composition.

## 2. Materials and Methods

### 2.1. Information Sources and Search Strategy

This systematic review was based on the updated Preferred Reporting Items for Systematic Reviews and Meta-Analyses (PRISMA) 2020 guidelines [21]. The literature search of the systematic review was carried out on 18 March 2022 in the PubMed, Scopus, and Cochrane databases using the following keywords: (((healthy individual* OR human* OR obes*) NOT (disease* OR disorder* OR syndrome OR diabetes OR cancer)) AND (“gut microbio*” OR “intestinal microbio*” OR “fecal microbio*” OR “cecal microbio*” OR microflora OR “gut bacteria” OR “intestinal bacteria”)) AND (“body composition” OR “fat-free mass” OR “fat mass” OR “body fat” OR “body mass” OR “body mass index” OR BMI OR “energy expenditure” OR “basal metabolic rate” OR BMR OR “resting metabolic rate” OR RMR). A supplementary search for relevant studies was conducted from the reference lists of the screened manuscripts.

### 2.2. Eligibility Criteria

The research question and inclusion and exclusion criteria were determined using the PICO strategy (Patient, Intervention, Controls, Outcome). The inclusion criteria were (1) primary research; (2) studies that presented the gut microbiome in the large intestine; (3) studies written in the English language; (4) studies that had as a population healthy children, adults, older adults, and postmenopausal women; (5) studies that intervened by providing probiotic, prebiotic, and symbiotic supplements; (6) studies that performed an intervention by modifying the diet or physical activity or both. The exclusion criteria were (1) non-primary research (i.e., reviews and case studies); (2) studies not written in English; (3) studies with a non-healthy population (except obese); (4) studies that presented the gut microbiome in other areas, such as the mouth; (5) studies that involved twins, infants, pregnancy, or breastfeeding; (6) studies that performed an intervention by providing medication. The PICO criteria for the inclusion and exclusion of studies are presented in Table 1.

### 2.3. Data Collection Process

Primary screening was conducted by two independent researchers (I.K., A.V.) using Microsoft Excel, according to the established eligibility criteria. Full-text secondary screening for the selection of the final articles was also conducted by these two independent researchers, while, where there were conflicts, a third independent researcher (CDG) resolved them.

### 2.4. Data Extraction and Quality Assessment

Data extraction from the final articles was conducted by one researcher (I.K.), who presented the results in four tables based on age groups (children, adults, older adults, and whole age range). Extracted data included the name of the first author and publication date, sample size, gender, age, BMI category, body composition, and results.

The quality assessment was conducted by one researcher using the Newcastle–Ottawa Scale (NOS) tool, adapted according to the study design [22]. The NOS tool consists of three sections regarding sample selection, a search for confounding factors, and study outcomes. As confounding factors, in the present systematic review, a check for the exclusion of antibiotic and/or probiotic intake was determined. The tool involves eight or nine questions and ten is the maximum score achieved. Due to the final score, studies were classified as “low quality” if the score was <5, as “moderate quality” if the score was 5–7, and as “high quality” if the score was >7.

Extracted data were categorized into four tables based on age groups: (i) children, <18 years, (ii) adults 18–65 years, (iii) older adults >65 years, and (iv) whole age range, children to older adults. The extracted data in each table were further categorized according to (1) sex—males and females; (2) BMI category—children were classified according to growth charts and adults according to BMI into (i) normo-weight, (ii) overweight, and (iii) obese, also considering the ethnicity-specific BMI cutoffs, as provided by the original articles; (3) body composition—some studies included information regarding body composition besides BMI, such as body fat percentage and muscle mass; (4) athletes—athletes were included in some studies as part of the sample to observe differences between them and non-athlete individuals. Athletes’ competition levels were determined based on the description provided by each study.

## 3. Results

### 3.1. Study Selection

**Figure 1:**
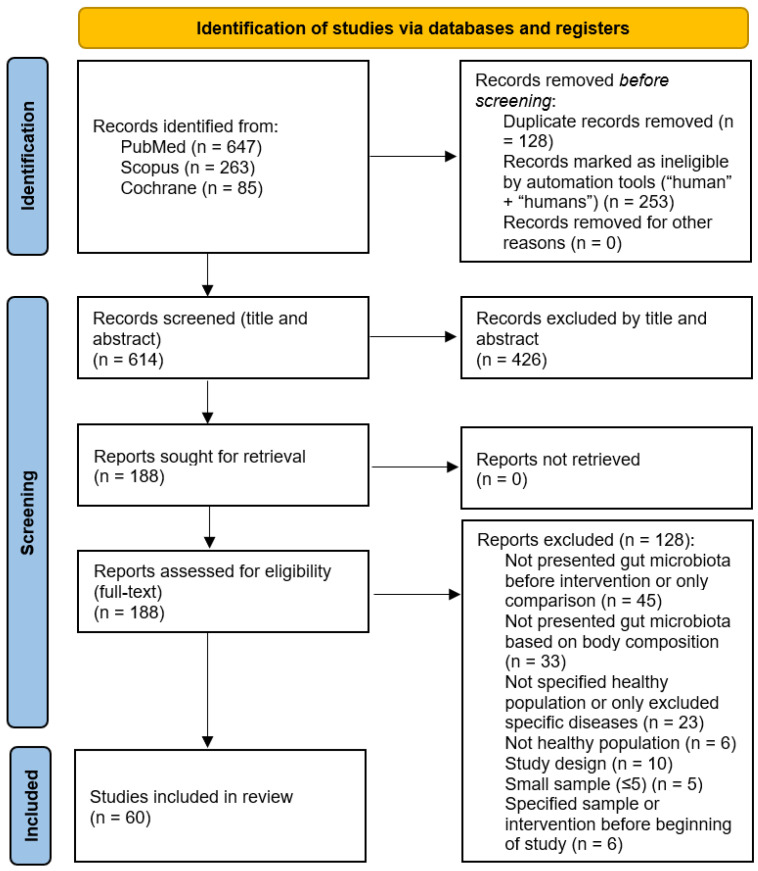
The Preferred Reporting Items for Systematic Reviews and Meta-Analyses (PRISMA) flow diagram for study selection.

During the search process using the keywords in the PubMed, Scopus, and Cochrane databases, 995 potentially relevant studies were found, with 614 studies remaining after duplicates were removed and the filters “human” and “humans” were applied. After the primary screening, which included the title and abstract reading, 188 studies were selected for full-text screening. The final studies that met the eligibility criteria and were included in this systematic review totaled 60. Figure 1 shows the study selection process in detail, according to the PRISMA 2020 guidelines.

### 3.2. Study Characteristics

The main characteristics of the studies included in this systematic review are presented in four categories based on age. Full summaries of the study characteristics, BMI categories, body composition, and results are provided in Table 2, Table 3, Table 4, Table 5 and Table 6. The gut microbiome was presented in all groups by stool collection, and, in the majority of the studies, the 16S rRNA amplicon sequencing method was applied [23,24,25,26,27,28,29,30,31,32,33,34,35,36,37,38,39,40,41,42,43,44,45,46,47,48,49,50,51,52,53,54], while the quantitative PCR (qPCR) method was applied to determine the bacterial abundance [16,25,27,30,31,33,42,44,47,48,50,54,55,56,57,58,59,60,61,62,63,64,65,66,67,68]. The body composition assessment was achieved by using WHO growth charts or BMI z-scores in children, while the BMI was used for both adults and older adults. Apart from the BMI, eight studies in children [23,24,27,31,55,58,60,69], 19 studies in adults [33,34,35,36,37,40,41,42,45,61,63,64,65,66,68,70,71,72,73], four studies in older adults [46,48,74], and one study in the whole age range [49] conducted further body composition measurements, such as the body fat percentage, visceral fat, lean body mass percentage, waist circumference, and waist/hip ratio.

The 18 studies with children as the target group were published between 2010 and 2022, with sample sizes ranging from 21 to 502 participants, while countries worldwide were included. None of the 18 studies included athletes. Fourteen studies were cross-sectional, one was a case–control study, and three were clinical trials. The 32 studies with adults as the target group were published between 2011 and 2021, with sample sizes ranging from 20 to 263 participants, while countries from all over the world were included. Three studies included athletes in their samples [34,39,64]. Nineteen studies were cross-sectional, one was longitudinal, 11 were clinical trials, and one was a comparative study. The four studies with older adults as the target group were published between 2017 and 2021, with sample sizes ranging from 22 to 201, with participants from Japan, Slovakia, and Italy. One study of this category included athletes in its sample [46]. Three studies were cross-sectional and one was a clinical trial. The six studies with participants from 14 to 88 years old were published between 2010 and 2021, with sample sizes ranging from 26 to 528, with participants from European and Asian countries. One study included athletes in its sample [49] and all six were cross-sectional.

The gut microbiome analysis was presented in each age group in terms of α-diversity, β-diversity, and bacterial taxonomy, including dominant phyla and genera and correlations of the gut microbiome composition with the BMI and other body composition measurements.

According to the Newcastle–Ottawa Scale (NOS), regarding evidence quality, two studies were rated as “low quality” (score < 5), all from the adult age group category [36,62]. Twenty-three studies were rated as “moderate quality” (score 5–7): eight from the children category [24,30,56,57,59,60,69,75], 11 from the adult category [33,35,37,38,39,42,61,63,73,76,77], three from the older adult category [47,48,74], and one from the whole age range category [53]. Thirty-five studies were rated as “high quality” (score > 7): 10 from the children category [23,25,26,27,28,29,31,32,55,58], 19 from the adult category [16,34,40,41,43,44,45,64,65,66,67,68,70,71,72,78,79,80,81], one from the older adult category [46], and five from the whole age range category [49,50,51,52,54].

**Table 3:** Characteristics of studies investigating the gut microbiome composition in adults.

Author(s), Date	N	Sex	Age (Years)	BMI Category (kg/m^2^)	Body Composition	Results
Allen et al., 2018 [61]	32	M and F	20–45	Lean: 22.21 ± 2.76 Obese: 35.71 ± 5.11	Lean (body fat % = 26.04 ± 6.12, lean mass % = 71.52 ± 6.18, bone density = 1.11 ± 0.08) Obese (body fat % = 38.42 ± 4.98, lean mass % = 59.42 ± 5.03, bone density = 1.21 ± 0.12)	Gut microbiota composition was different between lean and obese adults at baseline (*p* = 0.034)
Assmann et al., 2020 [33]	103	M and F	Eutropic: 44.7 ± 9.1 Obesity: 46.6 ± 9.4	Eutropic: 18.6 ± 2.1 Obesity: 32.9 ± 2.4	Eutropic (WC cm = 75.2 ± 7.6, fat mass % = 13.6 ± 5.7, lean mass % = 47.6 ± 12.2) Obesity (WC cm = 104.9 ± 10.2, fat mass % = 34.7 ± 6.5, lean mass % = 57.0 ± 11.7)	Bacterial genera: 18 were statistically different between obese and normal-weight individuals (*p* < 0.05) → ↑ *Mogibacterium*, *Mitsuokella*, *Megamonas*, *Howardella*, *Anaerovibrio*, *Bacteroides*, *Allisonella*, *Adlercreutzia*, *Abiotrophia*. ↓ *Victivallis*, *Succinivibrio*, *Rothia*, *Parvimonas*, *Intestimonas*, *Haemophilus*, *Faecalibacterium*, *Dorea*, *Anaerococcus* Bacterial species: 12 were statistically different between obese and normal-weight individuals (*p* < 0.02) → ↑ *Abiotrophia defectiva*, *Actinomyces odontolyticus*, *Allisonella histaminiformans*, *Barnesiella intestinihominis*, *Dorea longicatena*, *Howardella ureilytica*, *Lactobacillus curvatus*, *Megamonas funiformis*, *Mitsuokella jaladudinii*, *Odoribacter laneus*. ↓ *Bacteroides eggerthii*, *Haemophilus parainfluenzae*. Shannon index (α-diversity) was not different between obese and normal-weight groups. Β-diversity was statistically different.
Barnes et al., 2019 [62]	32	M and F	18–50	Lean control: 22.1 (1.6) Lean mango: 22.9 (2.2) Obese mango: 34.6 (4.9)	NR	Day 0: Obese → ↑ *Clostridium* leptum (*p* = 0.0264), *Bacteroides thetaiotaomicron* (*p* = 0.0359). ↓ *Lactococcus lactis* (*p* = 0.443).
Basciani et al., 2020 [63]	48	M and F	56.2 ± 6.1	Obese: 35.9 ± 4.1	WPG (WC = 110.0 ± 9.4 cm, HC = 123.6 ± 12.1 cm, TC = 63.6 ± 5.3 cm, arm circumference = 36.6 ± 3.9 cm) VPG (WC = 108.2 ± 8.5 cm, HC = 123.3 ± 9.3 cm, TC = 64.1 ± 5.3 cm, arm circumference = 36.3 ± 3.7 cm) APG (WC = 105.3 ± 9.1 cm, HC = 122.5 ± 10.6 cm, TC = 65.4 ± 7.2 cm, arm circumference = 37.7 ± 3.0 cm)	TO: Obese → dominant phyla: *Firmicutes*, *Bacteroidetes*, *Proteobacteria*, *Verrucomicrobia*, *Fusobacteria*, *Actinobacteria*. *Firmicutes*: 80–90%, *Bacteroidetes*: 0–10%.
Bezek et al., 2020 [70]	200	M and F	35.4 ± 7.0 (25–50)	24.2 ± 3.5 (18.5–35)	WHR: 0.87 ± 0.07, visceral fat index: 4.7 ± 2.9	All participants: Phylum (%) → *Firmicutes* (71.02 ± 11.45), *Bacteroidetes* (13.85 ± 10.20), *Proteobacteria* (3.52 ± 3.33), *Actinobacteria* (2.80 ± 3.25), *Verrucomicrobia* (0.28 ± 2.87). Genus (%) → *Blautia* (11.79 ± 5.84), *Faecalibacterium* (8.59 ± 5.09), *Bacteroides* (7.97 ± 8.05), *Ruminococcus* (6.51 ± 3.17), *Clostridium* (4.79 ± 3.48). Clusters (most prevalent): C1 → Phylum = *Bacteroidetes*, Genus = *Bacteroides*, *Prevotella*. C2 → Phylum = *Firmicutes*, Genus = *Blautia*, *Clostridium*. C3 → Phylum = *Actinobacteria*, Genus = *Bifidobacterium*. C4 → Phylum = *Proteobacteria*, *Verrucomicrobia*, Genus = *Erysipelothrix*. C2: higher obesity measures → ↑ *Firmicutes*, *Firmicutes*/*Bacteroidetes* (F/B) ratio, ↓ *Bacteroidetes*.
Bielik et al., 2020 [64]	24	M	Lean athletes (LA): 27.3 (23.5–31.0) Control athletes (CTRLs): 30.0 (25.1–34.9)	LA: 20.14 (19.31–20.97) CTRLs: 24.1 (22.9–25.2)	LA: body fat % = 11.73 (9.9–13.6) CTRLs: body fat % = 13.1 (11.2–14.9)	Phylum: *Actinobacteria* (*p* ≤ 0.01). Class: LA → ↓ *Gamma proteobacteria* (*Proteobacteria*) (*p* = 0.04), *Shewanella* (*p* = 0.04), *Xanthomonas* (*p* = 0.03). Order: LA → ↓ *Alteromonadales* (*Proteobacteria*) (*p* = 0.04). Genus: LA → ↑ *Roseburia* spp. (*Firmicutes*) (*p* = 0.03), *Barnesiella* spp. (*Bacteroidetes*) (*p* = 0.05). Family: LA → ↓ *Coriobacteriaceae* (*Actinobacteria*) (*p* = 0.04).
Bloemendaal et al., 2021 [78]	56	F	18–40	Probiotics group: 21.9 ± 0.32 Control group: 21.7 ± 0.30	NR	Phylum before intervention: *Firmicutes* (68.0%), *Bacteroidetes* (19.5%), *Actinobacteria* (8.7%), *Proteobacteria* (1.5%), *Verrucomicrobiota* (1.4%), *Euryarcheota* (0.4%), *Tenericutes* (0.29%), *Cyanobacteria* (0.25%).
Borgo et al., 2018 [71]	40	M and F	NW (M: 48.7 ± 10.2, F: 51.7 ± 8.3) O (M: 53.8 ± 7.7, F: 51.3 ± 6.7)	NW: 22.8 ± 1.8 O: 35.8 ± 8.3	NW (M: 83.1 ± 2.4, F: 82.9 ± 3.2) O (M: 112.1 ± 8.5, F: 109.3 ± 9.8)	Lumen-associated microbiota (LAM): Obese → ↓ α-diversity, *Oscillospira genus*. ↑ *Veillonellaceae*, *Dialister* spp. *Flavonifractor plautii* + *Faecalibacterium prausnitzii* negatively associated with BMI. Mucosal-associated microbiota (MAM): no significant differences between BMI groups.
Brignardello et al., 2010 [72]	24	M and F	18–50	Normal-weight: 23.5 ± 2.4 Obese: 35.9 ± 5.0	Normal-weight (waist circumference = 78.7 ± 7.5 cm, body fat = 25.1 ± 7.3%, fat body mass = 15.6 ± 3.8 kg, lean body mass = 47.2 ± 11.3 kg) Obese (waist circumference = 112.5 ± 9.6 cm, body fat = 48.9 ± 9.3%, fat body mass = 43.1 ± 11.2 kg, lean body mass = 54.9 ± 10.6 kg)	Obese: ↑ relative abundance of bacteria with 23–37% G + C content in their DNA, ↓ bacteria with 40–47% and 57–61% G + C content in their DNA. Dominant bacteria regarding G + C content: obese → 36.2 ± 1.0%, normal-weight → 41.7 ± 1.4%.
Clarke et al., 2014 [34]	86	M	Elite athletes: 28.8 ± 3.8 Low BMI controls: 28.1 ± 5.1 High BMI controls: 30.8 ± 5.6	Elite athletes: 29.1 ± 3.0 Low BMI controls: 22.7 ± 1.8 High BMI controls: 31.2 ± 3.0	Elite athletes (body mass = 101.3 ± 13.8 kg, body fat = 16.9 ± 6.1 kg, lean body mass = 80 ± 8.9 kg, waist/hip ratio = 0.8 ± 0.04) Low BMI controls (body mass = 74.3 ± 6.3 kg, body fat = 15 ± 4.6 kg, lean body mass = 55.4 ± 5.6 kg, waist/hip ratio = 0.8 ± 0.05) High BMI controls (body mass = 103.1 ± 13.8 kg, body fat = 33.9 ± 8.8 kg, lean body mass = 65 ± 8 kg, waist/hip ratio = 0.9 ± 0.07)	α-diversity: ↑ Elite athletes compared with both control groups, no difference between the control groups. Elite athletes—High BMI controls: ↑ 48 taxa (top 6 → *Firmicutes*, *Ruminococcaceae*, *S24-7*, *Succinivibrionaceae*, *RC9*, *Succinivibrio*), ↑ *Family Akkermansiaceae* (*p* = 0.049) + *Genus Akkermansia* (*p* = 0.035), ↓ *Bacteroidetes* (*p* = 0.022). Elite athletes—Low BMI controls: ↑ 40 taxa (top 6 → *Prevotellaceae*, *Erysipelotrichaceae*, *S24-7*, *Succinivibrionaceae*, *Prevotella*, *Succinivibrio*), ↓ *Lactobacillaceae* (*p* = 0.001), *Bacteroides* (*p* = 0.035), *Lactobacillus* (*p* = 0.001). High BMI controls—Low BMI controls: difference in 7 taxa, ↑ *Dorea* (*p* = 0.026), *Pseudobutyrivibrio* (*p* = 0.022), ↓ *Ruminococcaceae Incertae Sedis* (*p* = 0.021), *Akkermansia* (*p* = 0.006).
Dekker Nitert et al., 2020 [35]	36	M and F	No back pain: 34 (25–42) Back pain: 30 (27–36)	≥25. No back pain: 29.9 (28.0–32.4) Back pain: 30.9 (28.2–34.5)	No back pain: WHR = 1.1 (0.8–1.4) Back pain: WHR = 1.1 (0.9–1.2)	*Adlercreutzia*: positively correlated with BMI (*p* = 0.03).
Durk et al., 2019 [65]	37	M and F	25.7 ± 2.2 (22–32)	23.7 ± 3.6 (17.9–31.4)	Body fat % = 23.1 ± 9.1 (7.0–38.0), fat mass kg = 16.2 ± 8.0 (4.1–40.2), fat-free mass kg = 53.0 ± 11.4 (33.7–80.1)	F/B: statistically correlated only with VO_2_max (*p* < 0.003) No other BMI or body composition variables were significantly correlated.
F S Teixeira et al., 2013 [66]	32	F	Lean: 28.05 ± 6.9 Obese: 30.7 ± 5.7	Lean: 20.6–21.9 Obese: 32.8–36.7	Lean (waist circumference cm = 66.5–72.0, body fat % = 18.0–23.8) Obese (waist circumference cm = 89.5–97.0, body fat % = 36.7–38.9)	Obese: ↓ *Lactobacillus plantarum*, *Akkermansia muciniphila* (*p* = 0.06), *Bifidobacterium genus*, *Bifidobacterium longum*, *Clostridium coccoides*, *Clostridium leptum* (*p* < 0.05) → negative correlations with BMI and waist circumference (*p* < 0.05). Body fat %: correlated inversely with *Bifidobacterium genus*, *Bifidobacterium longum*, *Clostridium leptum*, *Clostridium coccoides*, *Lactobacillus plantarum* (*p* < 0.05).
Fernandes et al., 2014 [67]	94	M and F	LN: 32.0 ± 1.8 OWOB: 37.9 ± 2.0	LN: 21.8 ± 0.3 OWOB: 30.3 ± 0.7	NR	Obese: ↓ *Escherichia coli* (*p* = 0.005). F/B: not significantly different between 2 groups. Combined 2 groups: BMI → inversely related to *Bacteroidetes* (r = −0.21, *p* = 0.04) and *E. coli* (r = −0.34, *p* = 0.002), no association with F/B.
Gallè et al., 2020 [79]	140	M and F	22.5 ± 2.9 (18–36)	22.4 ± 2.8 (15.2–33.8)	NR	Phyla: 28 different phyla detected—the most abundant → *Firmicutes* (61.6 ± 14.6) and *Bacteroidetes* (30.7 ± 13.3). BMI (underweight/normal-weight—overweight/obese): No significant differences in Shannon index, *Firmicutes*, *Bacteroidetes*, and F/B. Genera → ↑ *Selemonas* (*p* = 0.02), *Megasphaera* (*p* = 0.001), *Streptococcus* (*p* = 0.001), *Dorea* (*p* = 0.001), *Lachnobacterium* (*p* = 0.007), *Jannaschia* (*p* = 0.02), *Dialister* (*p* = 0.001), *Eubacterium* (*p* = 0.01), ↓ *Paraprevotella* (*p* = 0.01) in overweight/obese compared with underweight/normal-weight participants.
Henning et al., 2019 [36]	63	M and F	CTRL: 36.4 ± 10.8 AVO: 42.5 ± 12.7	CTRL: 30.0 ± 3.7 AVO: 30.1 ± 3.2	CTRL: Total body fat % = 38.3 ± 8.5 AVO: Total body fat % = 41.2 ± 5.1	Baseline bacteria: Phylum (CTRL, AVO) → *Firmicutes* (61.29 ± 11.00, 53.91 ± 10.02), *Bacteroidetes* (26.94 ± 9.83, 34.88 ± 14.41), *Actinobacteria* (7.24 ± 6.07, 7.59 ± 7.86), *Euryarcheota* (1.76 ± 2.95, 1.05 ± 2.42), *Verrucomicrobia* (0.75 ± 1.90, 1.23 ± 1.73), *Proteobacteria* (1.09 ± 1.61, 0.89 ± 1.22). Family (CTRL, AVO)—Top 3 → *Bacteroidaceae* (*Bacteroidetes*) (17.27 ± 11.31, 23.37 ± 12.55), *Ruminococcaceae* (*Firmicutes*) (20.03 ± 6.02, 18.54 ± 7.33), *Lachnospiraceae* (*Firmicutes*) (16.56 ± 5.89, 15.37 ± 4.82). Genus (CTRL, AVO)—Top 3 → *Bacteroides* (*Bacteroidetes*) (17.27 ± 11.31, 23.37 ± 12.55), *Clostridium* (*Firmicutes*) (8.75 ± 3.17, 8.20 ± 3.41), *Dialister* (*Firmicutes*) (0.39 ± 0.61, 0.63 ± 1.01).
Hjorth et al., 2019 [37]	52	M and F	0-P: 47.9 ± 6.8 Low P/B: 43.4 ± 8.7 High P/B: 41.8 ± 11.5	0-P: 30.7 ± 1.1 Low P/B: 29.7 ± 2.2 High P/B: 31.9 ± 2.8	0-P: Body fat % = 48.7 ± 3.9 Low P/B: Body fat % = 44.9 ± 4.1 High P/B: Body fat % = 44.4 ± 5.0	Baseline: High P/B group → statistically significant ↑ body weight, BMI, relative abundance of *Prevotella* spp. and ↓ relative abundance of *Bacteroides* spp.
Janssens et al., 2016 [73]	58	M and F	Green tea: 28.2 ± 10.8 Placebo: 28.1 ± 10.5	Green tea: 23.0 ± 4.0 Placebo: 23.6 ± 4.6	Green tea (FMI kg/m^2^ = 6.9 ± 3.1, FFMI kg/m^2^ = 16.1 ± 1.9, WHR = 0.76 ± 0.09, FM kg = 19.9 ± 8.9, FFM kg = 46.9 ± 9.1, body fat % = 29.1 ± 8.2) Placebo (FMI kg/m^2^ = 7.2 ± 3.5, FFMI kg/m^2^ = 16.3 ± 2.0, WHR = 0.73 ± 0.08, FM kg = 20.4 ± 9.0, FFM kg = 47.2 ± 9.1, body fat % = 29.5 ± 8.7)	Participants categorized based on their BMI as normal-weight (18–25 kg/m^2^) and overweight (≥25 kg/m^2^). Baseline: Overweight → ↓ Shannon diversity index (α-diversity) for all phyla combined compared with normal-weight subjects (r = −0.39; *p* = 0.002).
Joller et al., 2020 [76]	26	F	25–35	30–35	NR	Baseline: 3 different enterotypes (most common to less common) → Enterotype 3—*Firmicutes*/*Ruminococcus* observed enriched in 21 females, Enterotype 2—*Prevotella* observed enriched in 3 females, Enterotype 1—*Bacteroides* observed enriched in 2 females. F/B ratio: ↑ (>1.6) in 12 females.
Kasai et al., 2015 [80]	56	M and F	N-Ob: 45.6 ± 9.6 Ob: 54.4 ± 8.2	Non-obese: BMI < 20 Obese: BMI ≥ 25	NR	Phylum: Obese → ↓ *Bacteroidetes*, ↑ F/B ratio, bacterial diversity and richness. Species: Obese → significantly associated with *Blautia hydrogenotorophica* (*Firmicutes*), *Coprococcus catus* (*Firmicutes*), *Eubacterium ventriosum* (*Firmicutes*), *Ruminococcus bromii* (*Firmicutes*), *Ruminococcus obeum* (*Firmicutes*); Non-obese → *Bacteroides faecichinchillae*, *Bacteroides thetaiotaomicron*, *Blautia wexlerae*, *Clostridium bolteae*, *Flavonifractor plautii*
Kobayashi et al., 2015 [38]	92	M	21–59	Lean: <18.5 Obese: >25.0 (17.3–30.2)	NR	*Bacillus* spp., *Erysipelothrix* spp., *Holdemania* spp. → related to lean group. *Microbacteriaceae*, *Actinobacterium* → related to obese group → Presence of *Firmicutes* and *Actinobacteria* may be related to BMI.
Koliada et al., 2017 [77]	61	M and F	20–60+	Underweight: <18.5 Normal: 18.5–24.9 Overweight: 25.0–29.9 Obese: ≥30	NR	Phylum: ↑ BMI → ↑ *Firmicutes*, F/B ratio, ↓ *Bacteroidetes*
Million et al., 2013 [16]	263	M and F	50 ± 17	Anorexic: 13.5 (11.7–14.6) Lean: 22.4 (20.7–23.7) Overweight: 27.1 (25.9–28.6) Obese: 40.0 (36.4–46.8)	NR	Positive correlation with BMI: *Lactobacillus reuteri* (*p* = 0.02). Negative correlation with BMI: *Bifidobacterium animalis* (*p* = 0.03), *Methanobrevibacter smithii* (*p* = 0.08), *Escherichia coli* (*p* < 0.001).
Most et al., 2017 [68]	37	M and F	37.8 ± 1.6	29.6 ± 0.5	EGCG + RES (waist/hip ratio = 0.88 ± 0.02, body fat % = 29.7 ± 1.9) F (waist/hip ratio = 0.87 ± 0.02, body fat % = 31.6 ± 1.4)	Baseline bacteria: Genus (PLA—EGCG + RES) → *Bacteroidetes* % (82.5 ± 2.9–84.3 ± 2.9), *Firmicutes* % (12.6 ± 2.1–12.5 ± 2.7), *Actinobacteria* % (2.8 ± 1–2 ± 0.5), *γ-Proteobacteria* % (1.7 ± 0.4–1.1 ± 0.3), *Akkermansia muciniphila* % (0.4 ± 0.2–0 ± 0). Males compared with Females → ↑ *Bacteroidetes* (*p* < 0.001), ↓ *Firmicutes* (*p* < 0.001), *Actinobacteria* (*p* = 0.04).
Murtaza et al., 2019 [39]	21	M	20–35	16.91–23.03	NR	Baseline bacteria: 3 distinct clusters (genus) → Cluster 1—*Prevotella dominant*, Cluster 2—*Bacteroides dominant*, Cluster 3—*Firmicutes dominant*. Cluster 1 and Cluster 2 were more common. Shannon diversity → no significant differences between 3 clusters.
Palmas et al., 2021 [40]	92	M and F	NW: 49 ± 11 OB: 50 ± 12	NW: 21.6 ± 2.1 OB: 36.0 ± 6.0	NW (waist circumference cm = 73.7 ± 5.7) OB (Fat mass kg = 39.1 ± 11.9, fat mass % = 42.3 ± 5.7, muscle mass kg = 48.5 ± 11.3, waist circumference cm = 111 ± 15)	Richness and diversity: α-diversity → ↓ in obese group, although no significant difference in Shannon index (*p* = 0.833). β-diversity → significant difference between 2 groups (*p* = 0.002). Bacterial abundance: Obese → ↑ F/B ratio (*p* = 0.007), *Firmicutes* and *Firmicutes taxa* (main biomarkers: *Lachnospiraceae*, *Megasphaera* spp. *+ Gemellaceae*, *Paenibacilleae*, *Streptococcaceae*, *Thermicanaceae*, *Gemella*, *Mitsuokella*, *Streptococcus*, *Acidaminococcus* spp., *Eubacterium* spp., *Ruminococcus* spp., *Megamonas* spp., *Streptococcus*, *Thermicanus*, *Veillonella* spp.), *Proteobacterium taxa* (main biomarkers: *Escherichia*, *E. albertii*), ↓ *Bacteroidetes* and *Bacteroidetes taxa* (main biomarkers: *Flavobacteria*, *Flavobacterium*, *Bacteroides* spp. *+ Porphyromonadaceae*, *Sphingobacteriaceae*, *Rikenella* spp., *Pedobacter* spp., *Parabacteroides* spp.). Body fat and waist circumference → negatively correlated with *Bacteroidetes taxa*. Body fat → positively correlated with *Firmicutes taxa*. Muscle mass and physical activity → negatively correlated with *Firmicutes taxa*.
Resende et al., 2021 [41]	24	M	20–45	CG: 23.68 ± 3.29 EG: 25.28 ± 4.11 (18.5–29.9)	CG (%FM = 21.87 ± 12.18, %FFM = 78.12 ± 12.18) EG (%FM = 23.59 ± 11.63, %FFM = 76.40 ± 11.63)	Baseline bacteria. 10 phyla were detected → most abundant: *Bacteroidetes*, *Firmicutes*, *Proteobacteria*—no statistical difference between 2 groups. BMI: negative correlation with *Desulfovibrio*. Body fat: negative association with *Faecalibacterium*. Fat-free mass %: positive association with *Faecalibacterium*.
Sergeev et al., 2020 [42]	20	M and F	Placebo: 47.0 ± 15.4 Synbiotic: 47.8 ± 8.99	Placebo: 32.77 ± 4.51 Synbiotic: 34.20 ± 5.60	Placebo (body mass kg = 97.6 ± 23.1, WC = 106.9 ± 12.47, body fat mass kg = 40.66 ± 6.92, body fat % = 40.97 ± 5.02, body lean mass kg = 57.39 ± 17.76, BMC kg = 2.66 ± 0.64, body lean mass + BMC kg = 60.05 ± 18.38) Synbiotic (body mass kg = 90.6 ± 11.9, WC = 109.6 ± 8.07, body fat mass kg = 36.97 ± 11.35, body fat % = 40.51 ± 8.96, body lean mass kg = 51.13 ± 8.87, BMC kg = 2.38 ± 0.48, body lean mass + BMC kg = 53.52 ± 9.35)	Baseline bacteria: *Firmicutes and Bacteroidetes* → the 2 most abundant phyla, *Bacteroides* → the most abundant genus.
Valeriani et al., 2020 [43]	59	M and F	23.1 ± 3.14 (20–36)	22.2 ± 2.6 (16.6–29.7)	NR	Phylum: Most abundant → *Firmicutes* (61.6 ± 14.6), *Bacteroidetes* (30.7 ± 13.3). Correlation analysis: BMI → positive but not significant correlation with *Firmicutes* (r = 0.22; *p* = 0.08), *Bacteroidetes* (r = 0.06; *p* = 0.63), F/B ratio (r = 0.11; *p* = 0.38).
Whisner et al., 2018 [44]	82	M and F	18.4 ± 0.6	<18.5 18.5–24.9 25.0–29.9 ≥30	NR	F/B ratio: 0.65 (0.39–1.23) → no statistically significant difference by BMI (*p* = 0.413).
Yang et al., 2017 [45]	71	F	19–49	Low VO_2_max: 31.7 (30.2–33.1) Moderate VO_2_max: 27.9 (26.7–29.1) High VO_2_max: 24.6 (23.0–26.2)	Low VO_2_max (fat % = 40.6 (38.1–43.0)) Moderate VO_2_max (fat % = 35.5 (33.2–37.8)) High VO_2_max (fat % = 28.0 (25.0–31.0))	Eubacterium rectale–*Clostridium coccoides*: positively correlated with fat% → ↑ in low VO_2_max, followed by moderate and high VO_2_max.
Zuo et al., 2011 [81]	104	M and F	Normal-weight: 33.02 ± 10.37 Obese: 34.65 ± 11.91	Normal-weight: 20.26 ± 1.50 (18.5–24) Obese: 30.79 ± 2.80 (≥28)	NR	Obese: ↓ *Bacteroides* (*p* = 0.012), *Clostridium perfringens* (*p* = 0.001). No other statistically significant differences in *Escherichia coli*, *Enterococci*, *Lactobacilli*, *Bifidobacteria* between groups → Enterococci: tendency to be ↑ in the obese group.

APG = Animal Protein Group; AVO = Avocado Group; BMI = Body Mass Index; CG = Control Group; CTRL = Control Group; EG = Exercise Group; F/B = Firmicutes to Bacteroidetes Ratio; F = Female; FFM = Fat-Free Mass; FM = Fat Mass; HC = Hip Circumference; LN = Lean; M = Male; NR = Not Reported; NW = Normal-Weight; O = Obese; OB = Obese; OW = Overweight; TC = Thigh Circumference; VO2 = Volume of Oxygen; VPG = Vegetable Protein Group; WC = Waist Circumference; WHR = Waist-to-Hip Ratio; WPG = Whey Protein Group.

**Table 4:** Characteristics of studies investigating the gut microbiome composition in older adults.

Author(s), Date	N	Sex	Age (Years)	BMI Category (kg/m^2^)	Body Composition	Results
Morita et al., 2019 [74]	29	F	70 (66–75)	21.4 (18.8–23.1)	Body fat % = 29.0 (23.6–32.7)	Baseline bacteria: Genus (TM group—AE group) → Bacteroides (40.7%–43.0%), Clostridium subcluster XIVa (16.6%–17.9%), Bifidobacterium (not available %), Clostridium cluster IV (not available %).
Šoltys et al., 2021 [46]	22	M	LA: 63.5 (61.4–65.7) CTRL: 64.9 (62.1–67.7)	LA: 24.8 (24.0–25.6) CTRL: 27.3 (24.9–29.7)	LA (total body fat % = 19.4 (17.3–21.5), visceral body fat = 9.5 (8.3–10.6), muscle mass % = 37.44 (34.9–40.0)) CTRL (total body fat % = 26.2 (21.9–30.5), visceral body fat = 14.1 (10.6–17.7), muscle mass % = 34.4 (27.6–44.9))	Dominant phylum (CTRL/LA): Firmicutes (73.9%/75.6%), Bacteroidetes (18.6%/14.4%), Proteobacteria (0.5%/1.5%). F/B ratio + α-diversity: no statistical difference between 2 groups. Family level: LA → ↑ Ruminococcaceae, ↓ Bacteroidaceae, Clostridiales Incertae Sedis XI, Cytophagia. Genus level: LA → ↑ Prevotella, Intestimonas, Subdoligranulum, Pseudobutyrivibrio, Marvinbryantia, Vallitalea, Porphyromonas, Anaerovorax, ↓ Bacteroides, Anaerosporobacter, Phascolarctobacterium, Bacteroides/Prevotella ratio.
Tamura et al., 2017 [47]	56	M and F	72.1 ± 0.6 (65–84)	23.1 ± 0.4	NR	Most abundant families: Lachnospiraceae (25.4% ± 1.3%), Ruminococcaceae (13.5% ± 1.0%), Bifidobacteriaceae (9.9% ± 1.2%), Streptococcaceae (6.0% ± 1.2%), Bacteroidaceae (5.9% ± 0.7%), Eubacteriaceae (4.9% ± 0.4%), Coriobacteriaceae (4.3% ± 0.5%), Peptostreptococcaceae (2.8% ± 0.5%), Enterobacteriaceae (2.0% ± 0.5%), Erysipelotrichaceae (1.7% ± 0.4%), Clostridiaceae (1.5% ± 0.3%), Lactobacillaceae (1.0% ± 0.2%), Porphyromonadaceae (0.8% ± 0.1%), Rikenellaceae (0.7% ± 0.1%), Prevotellaceae (0.6% ± 0.2%). Correlations between BMI and fecal microbiota: Negative correlations → Porphyromonadaceae (r = −0.342), Rikenellaceae (r = −0.299), Christensenellaceae (r = −0.341), Oxalobacteraceae (r = −0.329)—Positive correlations → Aerococcaceae (r = 0.32).
Tavella et al., 2021 [48]	201	M and F	71.2 ± 3.8 (65–79)	G1: 27.04 ± 3.60 G2: 24.68 ± 3.25 G3: 28.48 ± 4.18	G1 (waist circumference cm = 93.12 ± 11.63, hip circumference cm = 1014.3 ± 7.75, waist/hip ratio = 0.92 ± 0.09) G2 (waist circumference cm = 84.75 ± 9.31, hip circumference cm = 97.58 ± 7.36, waist/hip ratio = 0.86 ± 0.07) G3 (waist circumference cm = 95.79 ± 11.05, hip circumference cm = 104.75 ± 7.04, waist/hip ratio = 0.91 ± 0.08)	Overall: Most abundant phylum → Firmicutes (80%), Bacteroidetes (8.9%), Actinobacteria (7.4%). Most abundant family → Ruminococcaceae (37.5%), Lachnospiraceae (27.6%)—both belonging to Firmicutes). Most abundant genus → Subdoligranulum (12.5%), Faecalibacterium (7.8%), Bifidobacterium (4.6%). 3 groups: G1, G2, G3. α-diversity: ↑ G2, G3. G1 → enriched in Lachnospiraceae (Eubacterium rectale group, Fusitanetibacter, Blautia: negatively correlated with SMI—positively correlated with DXA variables, especially those related to fat mass distribution—FM, FMI, AF/AL, AF/GF, VAT) G2 (significantly ↓ anthropometric and body composition values) → enriched in Christensellaceae, Porphyromonadaceae, Rikenellaceae (Christensellaceae R7 group, Parabacteroides, Alistipes: inversely associated with DXA variables—visceral adipose tissue) G3 → enriched in Ruminococcaceae (Ruminococcaceae UCG 014, 002, 005: negatively correlated with most adiposity-related DXA variables, directly correlated with SMI and Faecalibacterium, Subdoligranulum, Ruminococcus: positively correlated with most adiposity-related DXA variables, negatively correlated with SMI).

AE = Aerobic Exercise Training; BMI = Body Mass Index; CTRL = Control; F = Female; LA: Lifetime Elderly Endurance Athletes; M = Male; NR = Not Reported; TM = Trunk Muscle Training.

**Table 5:** Characteristics of studies investigating gut microbiome composition regardless of age.

Author(s), Date	N	Sex	Age (Years)	BMI Category (kg/m^2^)	Body Composition	Results
Kulecka et al., 2020 [49]	71	M and F	14–72	NR	FMR (TBW lt = 30.9 ± 4.4, BF kg = 8.2 ± 1.1, FFM kg = 42.2 ± 5.9, MM kg = 23.4 ± 3.25) FCCS (TBW lt = 36.5 ± 2.7, BF kg = 9.3 ± 1.8, FFM kg = 50 ± 3.9, MM kg = 28.3 ± 2.3) MMR (TBW lt = 43.2 ± 3.6, BF kg = 5.9 ± 2.7, FFM kg = 59.8 ± 5.1, MM kg = 38.5 ± 10.1) MCCS (TBW lt = 49 ± 3.4, BF kg = 4.9 ± 1, FFM kg = 67 ± 4.74, MM kg = 39.3 ± 2.9)	Both athlete groups (MR, CCS) compared with healthy controls: ↓ Bacteroides, ↑ Prevotella, microbial diversity, and richness. F/B ratio: ↓ in healthy controls compared with CCS (*p* = 0.043), no statistically significant difference between healthy controls and MR.
La-Ongkham et al., 2020 [50]	120	M and F	Adult: 34.60 ± 3.19, elderly: 69.53 ± 3.44	Adult: 22.39 ± 3.33, elderly: 24.30 ± 2.68	NR	Phylum: >96% belonged to Firmicutes, Bacteroidetes, Proteobacteria, Actinobacteria. Statistically significant differences only in Bacteroidetes and Actinobacteria. Elderly → ↑ Bacteroidetes (phylum) (*p* = 0.019)—Bacteroidaceae (family) (*p* = 0.001)—Bacteroides (genus) (*p* = 0.001)—species: Bacteroides uniformis, Bacteroides ovatus, Bacteroides caccae, Bacteroides thetaiotaomicron, Parabacteroides (genus) (*p* = 0.02), ↓ Actinobacteria (phylum) (*p* = 0.001)—Bifidobacteriaceae (family) (*p* = 0.001)—Bifidobacterium (genus) (*p* = 0.001)—species: Bifidobacterium adolescentis, Bifidobacterium longum, Bifidobacterium pseudocatenulatum, Dorea (genus) (*p* = 0.01), F/B ratio (*p* = 0.01). ↑ age → ↓ Bifidobacterium, ↑ Bacteroides.
Latorre-Pérez et al., 2021 [51]	528	M and F	18.3–71	17.26–36.33	NR	All participants: Dominant phylum → Firmicutes (53.9%), Bacteroidetes (37.2%), Proteobacteria (5%), Verrucomicrobia (1.8%), Actinobacteria (0.9%). Dominant genera → Bacteroides (18.4%), Faecalibacterium (12.5%) (12.5%), Prevotella (6.7%), Alistipes (3.4%), Oscillospiraceae taxa (2.3%). ↑ BMI → positive correlation with Roseburia (genus), proteobacteria (phylum)—negative association with Marvinbryantia (genus) and Christensenellaceae (family). ↑ Age → ↓ Faecalibacterium, Bifidobacterium, ↑ alpha diversity—no significant associations with Akkermansia and Bacteroides
Martínez-Cuesta et al., 2021 [52]	26	M and F	18+	Normo-weight (N): 18–25, obese (O): >30	NR	Richness and diversity: Obese → ↓ Chao1 index (α diversity), no other statistical differences. Phylum: No statistical differences in Firmicutes, Bacteroidetes, F/B ratio. Family: Obese → ↓ Ruminococcaceae, Rikenellaceae, Peptostreptococcaceae, Clostridiales. Genus: Obese → ↑ Collisnella, Clostridium XIVa, Catenibacterium, ↓ Alistipes, Clostridium sensu stricto, Romboutsia, Oscilibacter.
Oki et al., 2016 [53]	516	M and F	52.4 ± 13.4 (21–88)	Lean: <25, obese: >30	NR	Predominant bacterial families: Bacteroidaceae (33.1 ± 19.0%), Lachnospiraceae (17.6 ± 10.1%), Ruminococcaceae (15.8 ± 9.3%), Prevotellaceae (9.1 ± 18.0%). Obese: ↓ Christensenellaceae, Mogibacteriaceae, Rikenellaceae (*p* < 0.05).
Schwiertz et al., 2010 [54]	98	M and F	47 ± 13 (14–74)	Lean: 18.5–24.9, overweight: 25.0–29.9, obese: ≥30.0	NR	Most abundant bacterial groups in all groups: Clostridium leptum group, Clostridium coccoides group, Bacteroides spp. → all belonged to Firmicutes and Bacteroidetes phyla. Differences between groups: Overweight/obese compared with lean → ↓ Firmicutes (*p* = 0.001, *p* = 0.002), F/B ratio (*p* = 0.001, *p* = 0.005), Ruminococcus flacefaciens subgroup (phylum: Firmicutes; *p* = 0.006, *p* = 0.011), ↑ Bacteroidetes (*p* = 0.001, *p* = 0.006). Overweight compared with lean → ↑ Bacteroides (*p* = 0.002). Obese compared with lean → ↓ Clostridium leptum group (*p* = 0.07), Bifidobacterium (*p* = 0.02), Methanobrevibacter (*p* = 0.017).

BF = Body Fat; BMI = Body Mass Index; CCS = Cross-Country Skiers; F/B Ratio = Firmicutes to Bacteroidetes Ratio; F = Female; FCCS = Female Cross-Country Skiers; FFM = Fat-Free Mass; FMR = Female Marathon Runners; M = Male; MCCS = Male Cross-Country Skiers; MM = Muscle Mass; MMR = Male Marathon Runners; MR = Marathon Runners; NR = Not Reported; TBW = Total Body Water.

**Table 6:** Main differences in gut microbiome composition in all BMI categories and age groups.

		α-diversity	Phyla			Genera (Phylum)												Species			
			Bacteroidetes	Firmicutes	Firmicutes/ Bacteroidetes Ratio	Akkermansia (Verrucomicrobia)	Alistipes (Bacteroidetes)	Bacteroides (Bacteroidetes)	Bifidobacterium (Actinobacteria)	Dorea (Firmicutes)	Eubacterium (Firmicutes)	Faecalibacterium (Firmicutes)	Intestimonas (Firmicutes)	Lactobacillus (Firmicutes)	Megasphaera (Firmicutes)	Oscilibacter (Firmicutes)	Streptococcus (Firmicutes)	Faecalibacterium Prausnitzii	Lactobacillus Plantarum	Akkermansia Muciniphila	*Roseburia* spp.
Children	Normo-weight	↑	↑	↓	↓	↑	–	↑	↑	↓	↓	–	–	–	–	↑	–	↓	↑	↑	–
	Overweight	↓	↓	↑	↑	↓	–	↓	↓	↑	↑	–	–	–	–	↓	–	↑	↓	↓	–
	Obese	↓	↓	↑	↑	↓	–	↓	↓	↑	↑	–	–	–	–	↓	–	↑	↓	↓	–
	Athletes	–	–	–	–	–	–	–	–	–	–	–	–	–	–	–	–	–	–	–	–
Adults	Normo-weight	↑	↑	↓	↓	–	↑	↑	↑	↓	↓	↑	↑	↓	↓	↑	↓	↑	↑	↑	–
	Overweight	↓	↓	↑	↑	–	↓	↓	↓	↑	↑	↓	↓	↑	↑	↓	↑	↓	↓	↓	–
	Obese	↓	↓	↑	↑	–	↓	↓	↓	↑	↑	↓	↓	↑	↑	↓	↑	↓	↓	↓	–
	Athletes	↑↑	↓	–	–	↑	–	–	–	–	–	–	–	–	–	–	–	–	–	–	↑
Older Adults	Normo-weight	–	–	–	–	–	–	–	–	–	–	–	–	–	–	–	–	–	–	–	–
	Overweight	–	–	–	–	–	–	↑	–	–	–	–	–	–	–	–	–	–	–	–	–
	Obese	–	–	–	–	–	–	↑	–	–	–	–	–	–	–	–	–	–	–	–	–
	Athletes	↑↓	–	–	↑↓	–	–	↓	–	–	–	–	↓	–	–	–	–	–	–	–	–

↑ = increased, ↓ = decreased, ↑↓ = contradictory, – = data not available.

### 3.3. Children

Most of the results from the studies in this category were presented comparing normal-weight with underweight or overweight children (Table 2). Regarding α-diversity, seven studies took it into consideration [23,24,26,27,28,29,57]; however, only two studies reported statistically significant differences (*p* < 0.05) in relation to body composition [23,28]. The first study identified three groups according to muscle mass [23], and the second one showed less α-diversity in obese children compared with normal-weight children [28]. Only one study included β-diversity, with statistically significant differences between obese and normal-weight children (*p* < 0.05) [28].

The dominant phyla identified throughout the studies, both in normal-weight and obese children, were, in descending order, *Firmicutes*, *Bacteroidetes*, *Proteobacteria*, *Actinobacteria*, and *Verrucomicrobia* [24,26,27,29,30,32]. Riva and colleagues identified the dominant families and genera. The dominant families were *Ruminococcaceae*, *Lachnospiraceae*, *Bacteroidaceae*, *Veillonellaceae*, *Bifidobacteriaceae*, *Prevotellaceae*, *Verrucomicrobiaceae*, *Rikenellaceae*, and *Christensellaceae*, while the dominant genera were *Bacteroides*, *Subdoligranulum*, *Faecalibacterium*, *Dialister*, *Bifidobacterium*, *Pseudobutyrivibrio*, and *Blautia* [29].

Correlations between the gut microbiome composition and BMI were observed in 15 studies [24,25,26,27,28,29,30,55,56,57,58,59,60,69,75] and can be summarized into six classification categories: phylum, class, order, family, genus, and species. At the phylum level, the composition of the gut microbiome in obese children comprised decreased *Bacteroidetes* and increased *Firmicutes*, *Actinobacteria*, and *Firmicutes*/*Bacteroidetes* ratios (F/B ratio), in comparison to normal-weight children [24,26,29,30,60,69]. At the class level, no study showed any results, while, at the order level, high levels of Pasteurellales were observed [32]. At the family level, obese children’s microbiomes were characterized by increased levels of *Lactobacillaceae*, *Enterobacteriaceae*, and *Lachnospiraceae* and decreased levels of *Bacteroidaceae*, *Porphyromonadaceae*, *Prevotellaceae*, *Desulfovibrio*, *Christensenellaceae*, and *Ruminococcaceae* [28,29,55,57]. At the genus level, the studies showed increased levels of *Blautia*, *Dorea*, *Eubacterium*, *Fusitanetibacter*, and *Bifidobacterium*, and decreased levels of *Bacteroides*, *Oscilibacter*, *Parabacteroidetes*, *Ruminococcus*, *Akkermansia*, and *Haemophilus* in obese children [24,26,29,57,69,75]. Finally, at the species level, increased levels occurred in *Faecalibacterium prausnitzii*, *Bacteroides fragilis group*, *Lactobacillus* spp., *Bacteroides eggerthii*, *Lachnospira*, and *Prevotella member* and decreased levels occurred in *Bifidobacterium* spp., *Akkermansia muciniphila*, *Bacteroides plebeius*, and species from *Christensenellaceae*, *Alistipes*, and the *Lactobacillus gasseri* subgroup [25,27,28,56,57,59].

Other body composition parameters, besides BMI were correlated with the gut microbiome composition in five studies [23,31,55,58,69]. More specifically, a positive correlation was observed between the phylum *Firmicutes* and the circumferences of the waist and head [58], while correlations were also observed between *Bacteroidetes* (*p* = 0.031; *p* = 0.012; *p* = 0.003), the F/B ratio (*p* = 0.075; *p* = 0.032; *p* = 0.002), *Actinobacteria* (*p* = 0.039; *p* = 0.053; *p* = 0.078), and visceral, subcutaneous, and hepatic fat [69]. A positive correlation was observed between the family *Lactobacillaceae* and visceral fat [55], while a correlation was also observed between the family *Ruminococcaceae* and the fat-free mass index (FFMI) Z-score in boys (*p* = 0.027) [31]. At the genus level, *Faecalibacterium* and *Lachnospira* were positively correlated with at least one of the following: ratio of total body lean soft tissue mass (TSM) to weight (TSMR), appendicular skeletal mass (ASM), ratio of appendicular skeletal mass to height (ASMI), and ASMI z-score. They were negatively correlated with at least one of the following: ratio of TSMR, total body lean soft tissue mass/total body fat (TSM/TBF), appendicular skeletal mass to weight (ASMR), appendicular skeletal mass/appendicular fat mass (ASM/AFM), and ASMR z-score [23]. The genera *Actinomyces*, *Bifidobacterium*, *Streptococcus*, and *Blautia* were positively correlated with body fat storage, while, in contrast, the genera *Odoribacter*, *Oscillospira*, *Bacteroides*, and *Faecalibacterium* were negatively correlated with fat [69].

### 3.4. Adults

The results from the studies in the adult category were presented by comparing normal-weight adults with either athletes or overweight/obese adults (Table 3). Five studies showed statistically significant differences in α-diversity (*p* < 0.05) [34,40,71,73,80], while two studies showed no differences (*p* > 0.05) [33,79]. More specifically, α-diversity was significantly lower in overweight/obese individuals, compared with the normal-weight control group (*p* < 0.05) [40,71,73], although Kasai and his colleagues reported the opposite result [80]. Clarke et al. [34] compared elite athletes with two groups of non-athletes, including both low and high BMI levels. Elite athletes showed statistically significantly higher levels of α-diversity compared with both groups, while no differences between the control groups were observed. Statistically significant differences between normal-weight and overweight individuals for β-diversity (*p* < 0.05) were observed in two studies [33,40].

The dominant phyla in all BMI adult groups in descending order were *Firmicutes*, *Bacteroidetes*, *Proteobacteria*, *Verrucomicrobia*, *Fusobacteria*, and *Actinobacteria* [36,41,42,43,63,68,70,78,79]. The three dominant families were *Bacteroidaceae*, *Ruminococcaceae*, and *Lachnospiraceae* [36]. The dominant genera were *Bacteroides*, *Clostridium*, *Dialister*, *Blautia*, *Faecalibacterium*, and *Ruminococcus*, all of which belong to the *Bacteroidetes* and *Firmicutes* phyla [36,42,68,70].

Correlations between the gut microbiome composition and BMI were observed from 20 studies [16,33,34,35,37,38,40,41,44,61,62,65,66,67,71,72,77,79,80,81], some of which did not show statistically significant differences [44,65]. The results were summarized into six classification categories, beginning with the phylum level. The composition of the gut microbiome in obese adults comprised increased levels of *Firmicutes*, increased F/B ratios, increased P/B ratios, and decreased levels of *Bacteroidetes* [38,40,65,67,80]. At the family level, *Ruminococcaceae*, *Succinivibrionaceae*, and *Akkermansia* were observed to be lower in obese adults, while *Microbacteriaceae* was higher [38]. At the genus level, obese adults had statistically significantly higher levels of *Mogibacterium*, *Mitsuokella*, *Megamonas*, *Howardella*, *Anaerovibrio*, *Allisonella*, *Adlercreutzia*, *Abiotrophia*, *Pseudobutyrivibrio*, *Adlercreutzia*, *Selemonas*, *Megasphaera*, *Streptococcus*, *Lachnobacterium*, *Jannaschia*, *Dialister*, *Eubacterium*, and *Actinobacterium* and lower levels of *Victivallis*, *Succinivibrio*, *Rothia*, *Parvimonas*, *Intestimonas*, *Haemophilus*, *Faecalibacterium*, *Anaerococcus*, *Paraprevotella*, and *Desulfovibrio* [33,34,35,40,41,71,79,81]. The results in the genera *Bacteroides* [33,40,81] and *Dorea* [33,34,79] were conflicting between studies. The *Bacteroides* levels [40,81] were decreased in obese adults, while, on the other hand, the *Dorea* levels [34,79] were elevated in the majority. Finally, at the species level, conflicting results were also observed for *Clostridium leptum* [62,66]. Otherwise, obese adults’ gut microbiomes were composed of increased levels of *Bacteroides thetaiotaomicron*, *Blautia hydrogenotorophica*, *Coprococcus catus*, *Eubacterium rentriosum*, *Ruminococcus bromii*, and *Lactobacillus reuteri*, most of which belong to the *Firmicutes* phylum, and decreased levels of *Lactococcus lactis*, *Flavonifractor plautii*, *Faecalibacterium prausnitzii*, *Lactobacillus plantarum*, *Akkermansia muciniphila*, *Bifidobacterium genus*, *Bifidobacterium longum*, *Bifidobacterium animalis*, *Clostridium coccoides*, *Clostridium perfringens*, *Escherichia coli*, *Bacillus* spp., *Erysipelothrix* spp., *Holdemania* spp., and *Methanobrevibacter smithii* [16,33,37,38,40,62,66,67,71,80,81].

Other body composition parameters, apart from BMI, were correlated with the gut microbiome composition in seven studies [34,40,41,45,65,66,70]. Obesity parameters, examples of which include the body fat percentage, visceral fat, waist circumference, and waist/hip ratio, were positively correlated with *Firmicutes*, the *Firmicutes* taxa, and the F/B ratio and negatively correlated with *Bacteroidetes* and the *Bacteroidetes* taxa, both in males and females (*p* < 0.05) [40,70]. A positive correlation was also observed in women between the body fat percentage and *Eubacterium rectale* and *Clostridium coccoides* [45]. On the other hand, another study showed a negative correlation between the waist circumference and *Clostridium leptum* (*p* < 0.05), as well as between body fat and *Bifidobacterium*, *Clostridium leptum*, and *Lactobacillus plantarum* in women (*p* < 0.05) [66]. A study conducted with a male sample showed a negative correlation between body fat and *Faecalibacterium* (*p* < 0.05) [41]. Both lean body mass and fat mass were negatively correlated with the *Firmicutes* taxa in males and females and positively correlated with *Faecalibacerium* in males (*p* < 0.05) [40,41]. Clarke et al. [34] compared elite male athletes with a control group of men with high BMI levels, who were not statistically significantly different from athletes. The two groups differed in the body fat percentage, lean body mass, and waist/hip ratio. Statistically significantly higher levels of the *Akkermansiaceae* family (*p* = 0.049) and the *Akkermansia* genus (*p* = 0.035) and lower levels of the *Bacteroidetes* phylum (*p* = 0.022) were observed in athletes [34]. Finally, one study did not show any statistically significant correlation between the gut microbiome composition and lean body mass or fat mass (*p* > 0.05) [65].

### 3.5. Older Adults

The results from the studies in the older adult category were presented by comparing older adults with different BMIs and long-term athletes versus sedentary control groups (Table 4). Two studies investigated α-diversity [46,48]; one did not show any statistically significant difference between the athlete group and the sedentary control group (*p* > 0.05) [46], while the other study categorized its sample into three groups according to different gut microbiome compositions and did show significant differences between groups (*p* < 0.05) [48]. None of the studies investigated β-diversity.

The dominant phyla in older adults were, in descending order, *Firmicutes*, *Bacteroidetes*, *Actinobacteria*, and *Proteobacteria* [46,48]. The three dominant families were *Lachnospiraceae*, *Ruminococcaceae*, and *Bifidobacteriaceae* [47,48]. The results on the genus level differed between studies. The dominant genera in older adults with lower BMI values (18.8–23.1 kg/m^2^) were *Bacteroides*, *Clostridium subcluster XIVa*, *Bifidobacterium*, and *Clostridium cluster IV* [74]. The dominant genera in older adults with higher BMI ranges were *Subdoligranulum*, *Faecalibacterium*, and *Bifidobacterium* [48].

Two out of four studies observed correlations between the gut microbiome composition and BMI [46,47]. Tamura et al. [47] showed a negative correlation between BMI and the families *Porphyromonadaceae* (r = −0.342), *Rikenellaceae* (r = −0.299), *Christensellaceae* (r = −0.341), and *Oxalobacteraceae* (r = −0.329) and a positive correlation between BMI and the family *Aerococcaceae* (r = 0.32). On the other hand, Soltys et al. [46] and her colleagues compared long-term athletes with a sedentary control group that had statistically significantly higher BMI values (*p* < 0.05). At the phylum level, the F/B ratio was not different between groups, while, at the family level, athletes had higher levels of *Ruminococcaceae* and lower levels of the *Bacteroidaceae*, *Clostridiales Incertae Sedis XI*, and *Cytophagia* families. Moreover, athletes had higher levels of the genera *Prevotella*, *Intestimonas*, *Subdoligranulum*, *Pseudobutyrivibrio*, *Marvinbryantia*, *Vallitalea*, *Porphyromonas*, and *Anaerovorax* and lower levels of *Bacteroides*, *Anaerosporobacter*, *Phascolarctobacterium*, and the *Bacteroides/Prevotella* ratio (*p* < 0.05).

In addition to BMI, other body composition parameters were correlated with the gut microbiome composition in two studies [46,48]. Soltys et al. [46], as described before, reported statistically significant differences between athletes and control groups in terms of the body fat percentage, visceral fat, and muscle mass percentage; these differences may have been responsible for the gut microbiome differences between the groups. The results of the second study were categorized into three groups according to the composition of the gut microbiome. The first group (G1) was enriched in the *Lachnospiraceae* family. The *Eubacterium rectale* group, *Fusitanetibacter*, and *Blautia* were negatively correlated with the skeletal muscle index (SMI) and positively correlated with the body fat distribution parameters (fat mass (FM), fat mass index (FMI), ratio of android fat mass/android lean mass (AF/AL), ratio of android fat mass/gynoid fat mass (AF/GF), visceral adipose tissue (VAT)). The second group (G2), with the significantly lowest anthropometric measurements, was enriched in the *Christensellaceae*, *Porphyromonadaceae*, and *Rikenellaceae* families. In the *Christensellaceae R7* group, *Parabacteroides* and *Alistipes* were negatively correlated with visceral fat. The last group (G3) was enriched in the *Ruminococcaceae* family. *Ruminococcaceae UCG 014*, *002*, and *005* were negatively correlated with body composition parameters referring to fat and positively correlated with the SMI. *Faecalibacterium*, *Subdoligranulum*, and *Ruminococcus* showed a reverse pattern compared to the above, with a positive correlation with body fat parameters and a negative correlation with the SMI (*p* < 0.05) [48].

### 3.6. Whole Age Range

The results from the studies in the whole age range category were presented by comparing people with different body composition measurements, regardless of age (Table 5). Three studies showed statistically significant differences for α-diversity (*p* < 0.05) [49,51,52]. The α-diversity was higher in athletes compared with non-athletes (*p* < 0.05) [49], older adults compared with adults (*p* < 0.05) [51], and normo-weight compared to obese individuals (*p* < 0.05) [52]. None of the studies investigated β-diversity.

The dominant phyla in all age groups were *Firmicutes*, *Bacteroidetes*, *Proteobacteria*, *Verrucomicrobia*, and *Actinobacteria* [50,51]. However, increasing age was observed to cause an increase in the Bacteroides and Bacteroides taxa and a decrease in the Actinobacteria and Actinobacteria taxa [50]. The dominant families were *Bacteroidaceae*, *Lachnospiraceae*, *Ruminococcaceae*, and *Prevotellaceae* [53]. The dominant genera were the *Bacteroides*, *Faecalibacterium*, *Prevotella*, *Alistipes*, and *Oscillosperaceae* taxa [51]. Finally, Schwiertz et al. [54] identified the most abundant bacterial groups, which were the *Clostridium leptum* group, *Clostridium coccoides* group, and *Bacteroides* spp., all belonging to the *Firmicutes* and *Bacteroidetes* phyla.

Three out of six studies described correlations between the gut microbiome and BMI [51,52,54]. The BMI was positively correlated with the *Roseburia* genus, while a negative correlation was found in the *Marvinbryantia* genus and *Christensellaceae* family [51]. Moreover, Martinez-Cuesta et al. [52] compared normo-weight with obese individuals. At the phylum level, no statistically significant correlation was observed in *Firmicutes*, *Bacteroidetes*, and the F/B ratio (*p* > 0.05). On the other hand, obese people had lower levels of the families *Ruminococcaceae*, *Rikenellaceae*, *Peptostreptococcaceae*, and *Clostridiales* and the genera *Alistipes*, *Clostridium sensu stricto*, *Romboutsia*, and *Oscilibacter* and higher levels of the genera *Collisnella*, *Clostridium XIVa*, and *Catenibacterium* (*p* < 0.05). Schwiertz et al. [54] compared normo-weight, overweight, and obese individuals. The gut microbiomes of overweight and obese individuals were found to have lower *Firmicutes* levels (*p* = 0.001, *p* = 0.002), F/B ratios (*p* = 0.001, *p* = 0.005), and *Ruminococcus flacefaciens* subgroup levels (*p* = 0.006, *p* = 0.011) and higher levels of *Bacteroidetes* (*p* = 0.001, *p* = 0.006). Overweight people had higher levels of *Bacteroides* (*p* = 0.002) and obese people had lower levels of the *Clostridium leptum* group (*p* = 0.07), *Bifidobacterium* (*p* = 0.02), and *Methanobrevibacter* (*p* = 0.017) compared with normal-weight individuals.

Correlations of the gut microbiome with other body composition parameters, besides BMI, were found only by Kulecka et al. [49]. The sample was categorized into three groups, marathon runners, skier athletes, and a sedentary control group. The body composition parameters, like body fat, lean body mass, and muscle mass, differed between the two athlete groups and the control group (*p* < 0.05). The results showed reduced levels of *Bacteroides* and increased levels of *Prevotella* in both athlete groups compared to the control group (*p* < 0.05). Increased levels of the F/B ratio were also observed in skiers compared with the control group (*p* = 0.043), while no statistically significant difference was observed in marathon runners (*p* > 0.05).

**Figure 2:**
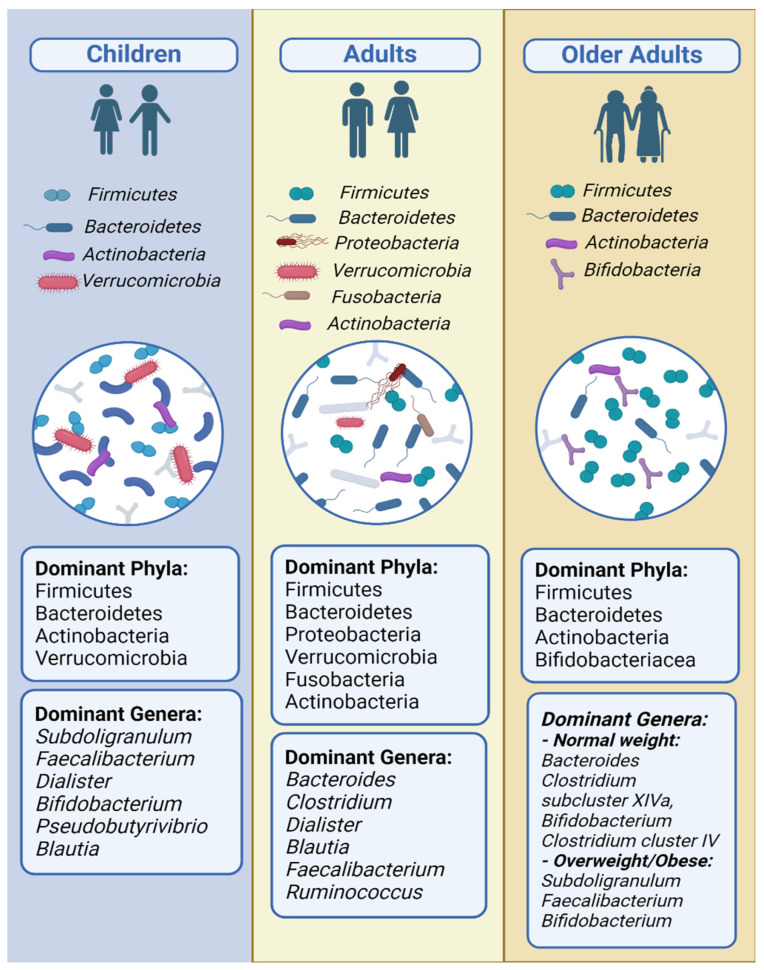
Predominant bacterial phyla and genera across distinct age groups.

**Figure 3:**
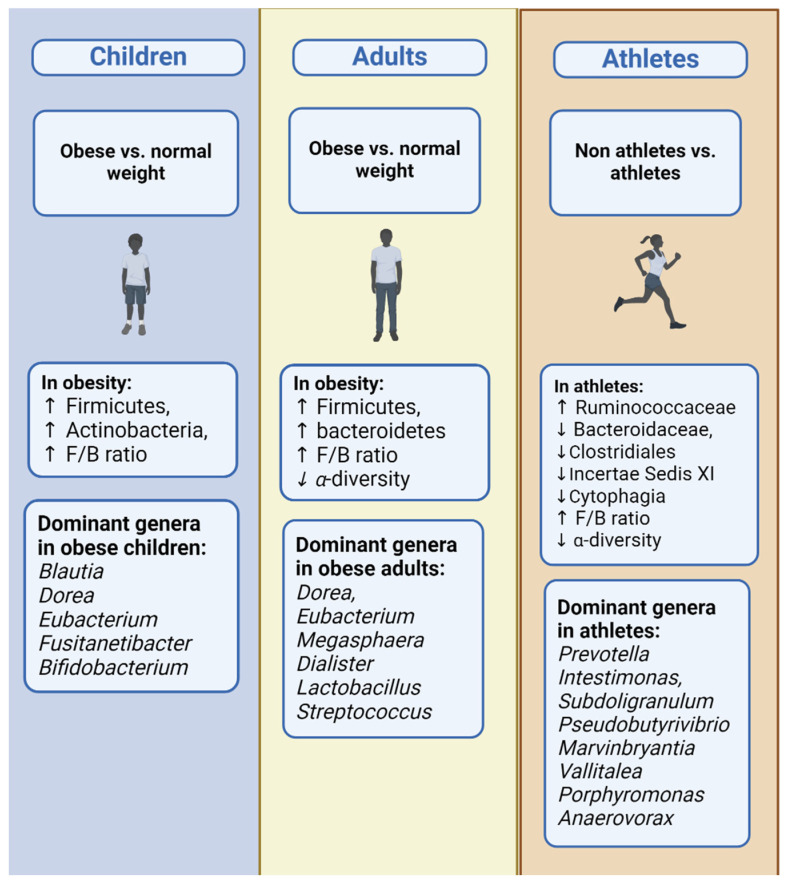
Bacterial phyla and general dynamics: contrasts between obesity and normal weight and across age groups including children, adults, and athletes.

The main differences in the gut microbiome composition in all BMI categories in all age groups are presented in Table 6. Figure 2 and Figure 3 show a comparative representation of the gut microbiome’s formation across the human lifespan. Children, adults, and older adults are categorized according to BMI into (i) normo-weight, (ii) overweight, (iii) obese, and (iv) athletes and are compared in terms of the gut microbiome composition regarding α-diversity and the most commonly found phyla, genera, and species.

## 4. Discussion

The present systematic review aimed to identify different gut microbiome profiles in healthy individuals, from children to older adults, and to correlate them with body composition formation. It was found that there are significant differences in the gut microbiome composition in individuals with excess weight or athletes across different age groups.

It was observed that the gut microbiome composition of overweight and obese participants was characterized by decreased α-diversity, mostly in adults compared to children, where only two [23,28] out of the seven studies [23,24,26,27,28,29,57] showed statistically significant differences. In addition, decreased levels of the *Bacteroidetes* phylum and its taxa and increased levels of the *Firmicutes* phylum, its taxa, and the F/B ratio were observed in comparison to normal-weight participants. Other body composition parameters, apart from the BMI, followed similar correlations. More specifically, a positive correlation between the *Firmicutes* phylum, its taxa, and obesity parameters, examples of which include the body fat mass and waist circumference, was observed, while a negative correlation was observed between the *Bacteroidetes* phylum, its taxa, and obesity parameters. On the other hand, the *Bacteroidetes* phylum and its taxa were also positively correlated with the lean body mass and muscle mass. These outcomes appeared to be more significant in athletes, even compared to normal-weight individuals.

The relationship between the gut microbiome composition and body weight has recently been discovered and continues to be studied widely, especially during the last decade [10,82]. Studies conducted in mice observed an alteration in body weight after a fecal transplant intervention from obese mice to mice without any microbiome; such an observation is responsible for the expanding studies conducted in humans [83]. The three main mechanisms through which the gut microbiome contributes to body weight are well known and have already been described in the Introduction of the current systematic review. Briefly, the first mechanism involves LPS promoting underlying inflammation, a common sign of obesity. The second mechanism involves the SCFAs that metabolize undigested food components like fiber, resulting in 10% more energy intake, while, in contrast, they contribute to other metabolic pathways, activating the secretion of anorexic hormones. The last mechanism involves bile acids, through which energy expenditure and the secretion of anorexigenic GLP-1 are promoted [8]. Despite the fact that the above mechanisms are well studied, the responsible bacteria are not yet fully identified [84].

According to the existing literature, the results for α-diversity between individuals with normal and excess weight are controversial. A meta-analysis conducted by Walters et al. [85] in 2014 did not show any statistically significant difference between normo-weight and overweight adults. In contrast, two more recent meta-analyses confirmed the reduced α-diversity in obesity observed in the current systematic review, although only two of the ten studies in Sze and Schloss’s meta-analysis showed statistically significant differences (*p* < 0.05) [86,87]. It is noteworthy that α-diversity is related to the better functionality of the gut microbiome; thus, a reduced α-diversity can lead to the disruption of the gut microbiome’s functioning and, ultimately, host dysbiosis [88]. Two recent systematic reviews examined the impact of exercise on α-diversity, confirming a positive association between α-diversity and individuals with high levels of fitness or cardiorespiratory fitness, as well as individuals with lower fitness levels after the impact of exercise [89,90].

At the phylum level, the F/B ratio, in the majority of the studies, was observed to be higher in obese compared with normo-weight individuals, in all age groups. However, two meta-analyses were in disagreement with our results, showing that the F/B ratio did not display statistically significant differences (*p* > 0.05) [15,91]. Thus, this measure cannot be considered a strong indicator for the separation of individuals based on BMI [87,92]. The phyla *Firmicutes* and *Bacteroidetes* are well known as the dominant phyla of the gut microbiome, making up over 90% of its composition [93]. The increased levels of the *Firmicutes* and decreased levels of *Bacteroidetes* observed in obese participants in the present systematic review are in agreement with a number of studies confirming the respective relationship [14,15,92]. More specifically, the phylum *Firmicutes* is positively correlated with parameters related to obesity, such as the body fat percentage, and negatively correlated with the lean body mass. In contrast, the phylum *Bacteroidetes* is negatively correlated with obesity parameters, a result that is also consistent with the present findings [19,85,94]. The observed relationship between *Firmicutes* and obesity parameters seems to be explained by the fact that many enzymes involved in carbohydrate metabolism belong to this phylum. The exact mechanism that promotes obesity is probably the one involving the production of SCFAs, as a positive correlation has been observed between the phylum *Firmicutes* and SCFAs in feces. This indicates that obese individuals prevail in the fermentation of undigested nutrients in the large intestine and, by extension, in the 10% excess energy production and in body weight gain [92,95,96,97,98]. Moreover, a second mechanism concerning SCFAs can explain the observed positive correlation between the phylum *Firmicutes* and body fat. The fermentation of fiber by SCFAs can also lead to the promotion of hepatic lipogenesis, increasing the storage and accumulation of fatty acids and triglycerides in the adipose tissue. Acetic acid is considered to be the main culprit responsible for this process and is mainly produced by bacteria belonging to *Firmicutes* [19,93].

Recent meta-analyses that investigated the gut microbiome’s composition in normo-weight and obese individuals confirm the results of the current systematic review at the genus level. Some commonly detected genera in obese individuals are increased levels of *Dorea*, *Eubacterium*, *Megasphaera*, *Dialister*, *Lactobacillus*, and *Streptococcus* (phylum *Firmicutes*) and decreased levels of *Bacteroides*, *Alistipes* (phylum *Bacteroidetes*), *Bifidobacterium* (phylum *Actinobacteria*), *Faecalibacterium*, and *Oscilibacter* (phylum *Firmicutes*). However, it is obvious that the relationship between the phylum level and obesity does not necessarily expand at the genus level. For instance, the genera *Faecalibacterium* and *Oscilibacter* are reduced in obese people, while the expected observation would be increased levels due to belonging to the phylum *Firmicutes* [15,85,99,100]. The exact mechanism through which some bacteria affect body weight has already been discovered. The bacteria *Lactobacillus plantarum*, *Faecalibacterium prausnitzii*, and *Akkermansia muciniphila* appear to be reduced in obese compared to normo-weight people, a correlation that was also found in the present study. The genus *Lactobacillus*, as a member of the phylum *Firmicutes*, has been associated with obesity and is found to be increased in those with excess weight. Some specific species, like *Lactobacillus plantarum*, have been shown to prevent dysbiosis through the production of bacteriocins that prevent the growth of pathogenic microorganisms [101]. *Faecalibacterium prausnitzii* causes the production of butyric acid from the fermentation of undigested nutrients and is also characterized by its anti-inflammatory role, explaining its protective role against obesity [102]. *Akkermansia muciniphila* participates in mucus metabolism and the maintenance of intestinal barrier integrity in the host, while it prevents the colonization of pathogenic microorganisms and dysbiosis [103].

As in every research study, there are some issues that need to be considered when interpreting the data of this review. Firstly, the majority of the studies included were cross-sectional; hence, their results do not reflect a cause–effect relationship. It is important to note that the prospective studies and clinical trials reported in this review included baseline data, before any intervention took place. Moreover, the heterogeneity between studies should also be considered, not only regarding the definition of obesity, which differs by country and age, but also regarding the level of bacterial taxonomy investigated by each study, making the comparison of the results difficult.

## 5. Conclusions

To conclude, the composition of the gut microbiome is evidently different in overweight individuals or athletes in all age groups. The composition of the gut microbiome in obese people comprises decreased α-diversity, decreased levels of the phylum *Bacteroidetes* and its taxa, and increased levels of the phylum *Firmicutes*, its taxa, and the F/B ratio. Besides the BMI, obesity parameters, like body fat mass, are positively correlated with the *Firmicutes* taxa and negatively correlated with the *Bacteroidetes* taxa, and lean fat mass and muscle mass are positively correlated with the *Bacteroidetes* taxa. Additional studies are needed to confirm the above results, including those with healthy older adults.

## Figures and Tables

**Table 1:** The PICO criteria for inclusion and exclusion of studies.

Parameter	Inclusion Criteria	Exclusion Criteria
Population	Healthy population, including children, adults, older adults, postmenopausal women	Non-healthy population, except obese Studies that involved twins, infants, pregnancy, breastfeeding
Intervention	Studies that presented the gut microbiome in the large intestine Studies that performed an intervention by providing probiotic, prebiotic, and symbiotic supplements Studies that performed an intervention by modifying diet or physical activity or both	Studies that presented the gut microbiome in other areas, such as the mouth Studies that performed an intervention by providing medication
Comparison	-	-
Outcome	Studies describing the results and differences in the gut microbiome composition in terms of body composition, such as BMI, fat mass, fat free mass, muscle mass	Studies that did not describe the results of the gut microbiome composition in terms of body composition
Type of publication	Primary research Studies written in the English language	Non-primary research, such as reviews and case studies Studies not written in English

**Table 2:** Characteristics of studies investigating the gut microbiome composition in children.

Author(s), Date	N	Sex	Age (Year)	BMI Category (kg/m^2^)	Body Composition	Results
Aguilar et al., 2020 [55]	93	M and F	8.4 ± 1.6	According to WHO criteria of BMI-for-age for children 5–19 years old. Normal-weight: −0.4 ± 0.7 Overweight: 1.5 ± 0.3 Obesity: 2.3 ± 0.3	Normal-weight (waist circumference cm = 55.9 ± 4.8, waist to height index = 0.4 ± 0, abdominal fat % = 21 ± 5, total body fat % = 25.7 ± 4.8) Overweight (waist circumference cm = 68.9 ± 8.1, waist to height index = 0.5 ± 0, abdominal fat % = 32.8 ± 6, total body fat % = 34.8 ± 4.8) Obesity (waist circumference cm = 74.3 ± 7, waist to height index = 0.6 ± 0, abdominal fat % = 38.3 ± 5.2, total body fat % = 39 ± 3.7)	Children with obesity and waist-to-height ratio < 0.5: ↓ *Bacteroidaceae*, *Porphyromonadaceae*, *Prevotellaceae* and ↑ *Lactobacillaceae*. Children with abdominal fat above median (>24%): ↑ *Lactobacillaceae*
Balamurugan et al., 2010 [56]	28	M and F	11–14	According to WHO reference growth charts. Non-obese: 1–85 percentile Obese: 97–99 percentile	NR	Obese: ↑ *Faecalibacterium prausnitzii* (*p* = 0.0253). No significant differences in *Bacteroides–Prevotella–Porphyromonas*, *Bifidobacterium*, and *Eubacterium rectale*.
Chen et al., 2022 [23]	412	M and F	6–9	LMM: 16.77 (3.14) MMM: 14.74 (1.91) HMM: 14.23 (1.69)	3 groups: low muscle mass (LMM), medium muscle mass (MMM), high muscle mass (HMM) LMM [TBF kg = 9.42 (5.00), TSM kg = 17.92 (5.11), TSMI kg/m^2^ = 10.59 (1.77), TSMR % = 63.23 (5.18), TSM/TFM % = 1.90 (0.48), ASM kg = 7.36 (2.51), ASMI kg/m^2^ = 4.29 (0.90), ASMR % = 25.65 (2.15), ASM/AFM % = 1.47 (0.38), ASMI Z-score = −0.49 (1.34), ASMR Z-score = −0.59 (0.70)] MMM [TBF kg = 5.87 (1.99), TSM kg = 16.70 (3.54), TSMI kg/m^2^ = 10.52 (1.25), TSMR % = 71.26 (3.69), TSM/TFM % = 2.85 (0.65), ASM kg = 6.78 (1.74), ASMI kg/m^2^ = 4.25 (0.63), ASMR % = 28.97 (1.91), ASM/AFM % = 2.31 (0.67), ASMI Z-score = −0.56 (1.17), ASMR Z-score = 0.51 (0.50)] HMM [TBF kg = 4.95 (1.63), TSM kg = 17.36 (3.36), TSMI kg/m^2^ = 10.80 (1.13), TSMR % = 74.91 (3.30), TSM/TFM % = 3.54 (0.72), ASM kg = 7.39 (1.88), ASMI kg/m^2^ = 4.57 (0.52), ASMR % = 31.96 (2.02), ASM/AFM % = 3.08 (0.80), ASMI Z-score = 0.02 (1.15), ASMR Z-score = 1.36 (0.49)]	α-diversity: statistically significant differences between 3 groups → Chao1 index: LMM-HMM (*p* = 0.0022), MMM-HMM (*p* = 0.0072), ACE: LMM-HMM (*p* = 0.0077), MMM-HMM (*p* = 0.011). β-diversity: significant difference between groups (*p* < 0.001). ↑ Genus: *Faecalibacterium*, *Lacnospira*, *Lachnospiraceae* → positively correlated ≥1 from TSMR, ASM, ASMI, ASMI Z-score, negatively correlated ≥1 from TSMR, TSM/TBF, ASMR, ASM/AFM, ASMR Z-score. No significant correlation in F/B ratio. Adjustment for TBF and BMI → Genus: statistically significant correlations only in *Faecalitalea* and *Pyramidobacter*.
Cho., 2021 [24]	60	M and F	Fat loss: 10.0 ± 2.4 Fat gain: 10.3 ± 2.7	Fat loss pre: 26.41 ± 4.04 Fat gain pre: 25.70 (23.75–27.30)	Fat loss pre (waist circumference = 88.90 [75.00–93.20] cm, waist-to-height ratio = 0.58 [0.54–0.61] cm, total body fat = 38.30 [35.60–43.0]%, skeletal muscle mass = 17.70 [13.90–21.80] kg, total body fat = 22.80 ± 7.89 kg, visceral fat = 112.10 [74.30–144.20] cm^2^, abdomen fat = 0.85 ± 0.08%) Fat gain pre (waist circumference = 88.81 ± 13.26 cm, waist-to-height ratio = 0.59 [0.55–0.62] cm, total body fat = 38.79 ± 5.16%, skeletal muscle mass = 17.80 [15.70–22.70] kg, total body fat = 21.60 [18.80–26.80] kg, visceral fat = 118.76 ± 49.54 cm^2^, abdomen fat = 0.86 ± 0.10%)	Baseline analysis. Phylum: Dominant bacteria in both groups → *Firmicutes*, *Bacteroidetes*, *Proteobacteria*, *Actinobacteria*, *Verrucomicrobia*. Fat gain group → ↓ *Bacteroidetes* compared with control group. Genus: Both groups → ↑ *Blautia*, *Dorea*, *Eubacterium hallii*, *Fusicatenibacter* compared with control group. Fat gain group → ↓ *Bacteroides*, *Oscillibacter*, *Parabacteroides*. Shanon diversity index: no significant difference between both preintervention groups and control group.
Goffredo et al., 2016 [69]	84	M and F	12.4 ± 2.9	Non-obese: BMI < 85th Overweight: 85th < BMI < 95th Obese: 95th < BMI < 99th Severely obese: BMI > 99th	Lean (body fat % = 20.62 ± 5.69, visceral body fat cm^2^ = 20.17 ± 11.18, SC cm^2^ = 153.79 ± 87.07, hepatic fat content % = 1.26 ± 1.81) Overweight (body fat % = 31.07 ± 5.59, visceral body fat cm^2^ = 36.60 ± 18.12, SC cm^2^ = 313.90 ± 12.87, hepatic fat content % = 0.466 ± 1.09) Obese (body fat % = 41.31 ± 7.16, visceral body fat cm^2^ = 57.44 ± 23.79, SC cm^2^ = 434.86 ± 164.21, hepatic fat content % = 9.16 ± 11.36) Severely obese (body fat % = 48.48 ± 9.11, visceral body fat cm^2^ = 79.31 ± 30.74, SC cm^2^ = 648.19 ± 214.20, hepatic fat content % = 13.00 ± 14.33)	Phylum: Total bacterial load → no association with body composition. F/B (*p* = 0.016), *Actinobacteria* (*p* = 0.01) → positively associated with BMI. Bacteroidetes (*p* = 0.0003) → inversely associated with BMI. F/B (*p* = 0.075; *p* = 0.032; *p* = 0.002), *Bacteroidetes* (*p* = 0.031; *p* = 0.012; *p* = 0.003), *Actinobacteria* (*p* = 0.039; *p* = 0.053; *p* = 0.078) → associated with visceral fat, SC fat and hepatic fat content. Genera: *Actinomyces*, *Bifidobacterium*, *Streptococcus*, *Blautia* → positively correlated with obesity and body fat deposits. *Odoribacter*, *Oscillospira*, *Bacteroides*, *Faecalibacterium* → inversely correlated with adiposity.
Ignacio et al., 2016 [25]	84	M and F	Lean: 6.1 ± 2.4 Overweight: 8.0 ± 2.0 Obese: 8.5 ± 2.6	Lean: BMI z-score 0.19 ± 0.72, Overweight: BMI z-score 1.68 ± 0.33, Obese: BMI z-score 3.5 ± 1.6	NR	Obese + overweight compared with lean: ↑ *Bacteroides fragilis* group (*p* = 0.015), *Lactobacillus* spp. (*p* = 0.022), ↓ *Bifidobacterium* spp. (*p* = 0.042), no significant difference in *Clostridium Cluster I*, *Methanobrevibacter smithii*, *E. coli*. BMI: positive correlation with *B. fragilis* group (r = 0.24; *p* = 0.026) and *Lactobacillus* spp. (r = 0.44; *p* = 0.002), negative correlation with *Bifidobacterium* spp. (r = −0.22; *p* = 0.039).
Karlsson et al., 2012 [57]	40	M and F	OO group: 4.67 (4.17–5.17) C group: 4.70 (4.33–4.98)	OO group: 20.55 (18.78–21.90) C group: 15.54 (14.98–16.07)	NR	OO group: ↑ *Enterobacteriaceae* (*p* = 0.036), ↓ *Desulfovibrio* (*p* = 0.027), *Akkermansia muciniphila* (*p* = 0.030). No statistical differences in *Lactobacillus* (*p* = 0.947), *Bifidobacterium* (*p* = 0.821), *Bacteroides fragilis* group (*p* = 0.104). Diversity → less diverse (not statistically significant; *p* = 0.091)
Karvonen et al., 2019 [26]	502	M and F	3	Overweight/obese: >85th percentile Non-overweight/non-obese: <85th percentile	NR	Phylum: Most abundant → *Firmicutes* (62.4%) and *Bacteroidetes* (24.2%) → No statistical differences between 2 groups. F/B ratio → no statistical differences. Genus: Overweight/Obese → ↑ *Dorea*, ↓ *Ruminococcus*, *Akkermansia*, *Parabacteroidetes*. Diversity: No associations between the groups.
Leong et al., 2020 [75]	319	M and F	5	Normal: BMI z-score < 1.036 Overweight/obese: BMI z-score ≥ 1.036	NR	PCs—genera: PC1 → negative loadings of *Christensellaceae*, *Ruminococcaceae*. PC2 → negative loadings of Bacteroides—positive loadings of *Bifidobacterium*, *Fusitanetibacter*. PC3 → positive loadings of *Faecalibacterium*, *Eubacterium*, *Roseburia*. Only PC1 and PC2 statistically correlated with BMI z-score → PC1 with ↓ BMI z-score and PC2 with ↑ BMI z-score. No statistical correlations observed between PC3 and F/B ratio and BMI z-score.
López-Contreras et al., 2018 [27]	138	M and F	6–12	NW: BMI percentile % = 39.27 ± 13.51 Obese: BMI percentile % = 96.92 ± 1.33	NW: Body fat % = 24.53 ± 6.60 Obese: Body fat % = 44.6 ± 5.41	Most abundant phylum in 2 groups (NW—Obese): *Bacteroidetes* (67.5%, 69.4%), *Firmicutes* (27.8%, 26%), *Proteobacteria* (3.4%, 3.5%). NW—Obese: no significant differences from phyla to genus, F/B ratio, richness, alpha diversity. Species: Obese → ↑ *Bacteroides eggerthii* (q = 0.004), ↓ *Bacteroides plebeius* (q = 0.046), unclassified species from *Christensenellaceae* family (q = 0.061).
McCann et al., 2021 [28]	54	M and F	Healthy weight controls (HWC): 15.0 ± 1.7 Obese (OB): 12.6 ± 2.4	HWC: BMI percentile % = 75.6 ± 2.9 OB: BMI percentile % = 137.8 ± 48.7	NR	α- and β-diversity → significantly different between 2 groups. Obese: ↓ *Christensellaceae* (family), *Ruminococcaceae* (family), *Alistipes* (species) *Bacteroides* family members, ↑ *Lachnospiraceae* (family), *Lachnospira* (species), *Prevotellaceae* members.
Miranda et al., 2019 [58]	96	F	14–19	G1: EUT + adequate BF% G2: EUT + high BF% G3: OW or OB + high BF%	G1 (WC: 61.0–67.2, WtHR: 0.38–0.41, NC: 28.0–30.0, Android fat %: 9.8–16.5, Gynoid fat %: 30.6–36.7) G2 (WC: 68.1–75.3, WtHR: 0.42–0.46, NC: 29.2–31.0, Android fat %: 17.9–30.5, Gynoid fat %: 37.9–46.9) G3 (WC: 78.7–88.2, WtHR: 0.48–0.53, NC: 31.0–34.0, Android fat %: 30.5–46.8, Gynoid fat %: 45.5–54.1)	Phylum: No significant differences in *Firmicutes*, *Bacteroidetes*, *Proteobacteria* between 3 groups *Firmicutes* → positively associated with WC and NC, but not with BMI and BF%.
Nagata et al., 2017 [59]	34	M and F	Obese: 10.8 ± 4.4 Control: 8.5 ± 2.9	Control: BMI Z-score = 0.1 ± 0.7 Obese: BMI Z-score = 2.7 ± 1.7 (>2.0)	NR	Baseline analysis. Obese (compared with controls): ↓ Total bacteria (8.9 ± 1.3–10.6 ± 0.2 Log10 cells/g; *p* < 0.05), *Bacteroides fragilis* group (8.5 ± 1.1–9.8 ± 0.4 Log10 cells/g; *p* < 0.05), *Bifidobacterium* (7.9 ± 1.5–9.8 ± 0.5 Log10 cells/g; *p* < 0.001), *Atopobium* cluster (7.7 ± 0.8–9.0 ± 0.7 Log10 cells/g; *p* < 0.05), *Lactobacillus gasseri* subgroup (4.4 ± 1.8–5.0 ± 1.4 Log10 cells/g; *p* < 0.05).
Riva et al., 2017 [29]	78	M and F	Normal-weight (N): 11 ± 0.33 Obese (O): 11 ± 1.99	According to WHO criteria. N: BMI z-score = 0.3 ± 0.82, O: BMI z-score = 3.0 ± 0.7	NR	Phylum: Predominant bacteria in both groups → *Bacteroides*, *Firmicutes*, *Actinobacteria*, *Verrucomicrobiota*, *Proteobacteria*. Family: Most abundant in both groups → *Ruminococcaceae*, *Lachnospiraceae*, *Bacteroidaceae*, *Veillonellaceae*, *Bifidobacteriaceae*, *Prevotellaceae*, *Verrucomicrobiaceae*, *Rikenellaceae*, *Christensellaceae*. Genus: Most abundant in both groups → *Bacteroides*, *Subdoligranulum*, *Faecalibacterium*, *Dialister*, *Bifidobacterium*, *Pseudobutyrivibrio*, *Blautia*. Obese children: Phylum → ↑ *Firmicutes* (N: 60.9 ± 14.1, O: 72.1 ± 12.1), F/B ratio (N: 2.6 ± 1.83, O: 7.7 ± 7.1; *p* < 0.001), ↓ *Bacteroidetes* (N: 30 ± 12.6, O: 16.6 ± 11.8). Family → ↑ *Ruminococcaceae* (N: 33.3 ± 11.5, O: 42.5 ± 12.7), ↓ *Bacteroidaceae* (N: 21.4 ± 12.2, O: 10 ± 7.1). Genus → ↓ *Bacteroides* (N: 21.4 ± 12.2, O: 10.5 ± 7.1). No significant differences → members of *Ruminococcaceae*, gut microbiota richness (*p* = 0.59), α-diversity (*p* = 0.34). BMI z-score → positively correlated with *Firmicutes*, *Ruminococcaceae*, and *Faecalibacterium prausnitzii* and negatively correlated with *Bacteroidetes*, *Bacteroidaceae*, and *Bacteroides*.
Ruiz et al., 2017 [30]	21	M and F	14.8 (13–16)	Lean: 21.8 (17.94–23.56) Obese: 32.2 (25.35–38.34)	NR	Baseline. Dominant bacteria in both groups → *Firmicutes*, *Bacteroidetes*, *Proteobacteria*, *Actinobacteria*, *Verrucomicrobia*. Obese → ↑ *Firmicutes*, F/B ratio, *Actinobacteria*, ↓ *Bacteroidetes*
Smith-Brown et al., 2018 [31]	36	M and F	2.65 (2.24–3.13)	BMI Z-score = 0.54 ± 0.78	FMI Z-score = 0.86 ± 1.46, FFMI Z-score = −0.54 ± 1.03, WHR Z-score = 0.49 ± 0.92	Microbiota composition significantly associated with FFMI Z-score in boys (*p* = 0.027), but not girls (*p* = 0.553) → FFMI Z-score in boys: significantly correlated with *Ruminococcaceae* (family). FFMI Z-score of well-nourished boys: positively associated with *Dorea formicigenerans*, *Faecalibacterium prausnitzii*, *negatively* associated with *Bacteroides cellulosilyticus*.
Xu et al., 2012 [60]	175	M and F	9.87 ± 1.97	Normal group: 16.53 ± 1.69 Overweight group: 20.14 ± 1.83 Obesity group: 24.94 ± 3.11	Normal group (waist cm = 58.27 ± 4.9, hip cm = 70.26 ± 6.65) Overweight group (waist cm = 65.08 ± 6.75, hip cm = 76.04 ± 8.7) Obesity group (waist cm = 76.72 ± 9.22, hip cm = 87.52 ± 12.41)	Phylum: Obesity group → ↓ *Bacteroidetes* compared with normal group (*p* = 0.002), F/B ratio compared to both normal and overweight group (*p* < 0.001)—no statistically significant difference in *Firmicutes* → negative correlation between BMI and *Bacteroidetes* (r = −0.18; *p* = 0.017), negative correlation between BMI and F/B ratio (r = −0.22; *p* = 0.003). Gender differences: Normal-weight girls → ↑ *Bacteroidetes* compared with normal-weight boys (*p* < 0.05) and compared with obese girls (*p* = 0.002)—no statistically significant differences between normal-weight and obese boys.
Yuan et al., 2020 [32]	89	M and F	Non-puberty: 8.36 ± 1.64 Puberty: 10.99 ± 1.15	Non-puberty: BMI z-score = 1.92 ± 1.79 Puberty: BMI z-score = 2.01 ± 1.13	NR	Core microbiota: Dominated by *Firmicutes*, *Bacteroidetes*, *Proteobacteria* in both groups. Non-puberty group: ↑ *Clostridiales* (order), *Pasteurellales* (order), *Clostridiaceae* (family), *Coprobacillus* (genus), *Haemophilus* (genus). Puberty group: ↑ *Betaproteobacteria* (class), *Burkholderiales* (order). Correlations with BMI z-score: positive correlations with *Pasteurellales* (order) (r = 0.223; *p* = 0.036), Haemophilus (genus) (r = 0.222; *p* = 0.036)—no other statistically significant correlations.

AFM = Appendicular Fat Mass; ASM = Appendicular Skeletal Mass; ASMI = Ratio of ASM to Height; ASMR = Ratio of ASM to Weight; BF = Body Fat; BMI = Body Mass Index; F = Female; HMM = High Muscle Mass; LMM = Low Muscle Mass; M = Male; MMM = Medium Muscle Mass; NC = Neck Circumference; NR = Not Reported; NW = Normal-Weight; TBF = Total Body Fat; TFM = Total Fat Mass; TSM = Total Body Lean Soft Tissue Mass; TSMI = Ratio of TSM to Height; TSMR = Ratio of TSM to Weight; WC = Waist Circumference; WHO = World Health Organization; WtHR = Waist-to-Height Ratio.
